# Ultrasound Neuromodulation of the Spleen Has Time-Dependent Anti-Inflammatory Effect in a Pneumonia Model

**DOI:** 10.3389/fimmu.2022.892086

**Published:** 2022-06-16

**Authors:** Umair Ahmed, John F. Graf, Anna Daytz, Omar Yaipen, Ibrahim Mughrabi, Naveen Jayaprakash, Victoria Cotero, Christine Morton, Clifford Scott Deutschman, Stavros Zanos, Chris Puleo

**Affiliations:** ^1^ Institute of Bioelectronic Medicine, Feinstein Institutes for Medical Research, Manhasset, NY, United States; ^2^ General Electric Research, Niskayuna, NY, United States

**Keywords:** neuro-immune communication, cholinergic anti-inflammatory pathway, ultrasound, neuromodulation, infection, diagnosis, infectious disease, pneumonia

## Abstract

Interfaces between the nervous and immune systems have been shown essential for the coordination and regulation of immune responses. Non-invasive ultrasound stimulation targeted to the spleen has recently been shown capable of activating one such interface, the splenic cholinergic anti-inflammatory pathway (CAP). Over the past decade, CAP and other neuroimmune pathways have been activated using implanted nerve stimulators and tested to prevent cytokine release and inflammation. However, CAP studies have typically been performed in models of severe, systemic (e.g., endotoxemia) or chronic inflammation (e.g., collagen-induced arthritis or DSS-induced colitis). Herein, we examined the effects of activation of the splenic CAP with ultrasound in a model of local bacterial infection by lung instillation of 10^5^ CFU of Streptococcus pneumoniae. We demonstrate a time-dependent effect of CAP activation on the cytokine response assay during infection progression. CAP activation-induced cytokine suppression is absent at intermediate times post-infection (16 hours following inoculation), but present during the early (4 hours) and later phases (48 hours). These results indicate that cytokine inhibition associated with splenic CAP activation is not observed at all timepoints following bacterial infection and highlights the importance of further studying neuroimmune interfaces within the context of different immune system and inflammatory states.

## Introduction

Communication between the nervous and immune systems can significantly alter immune cell function and response to inflammatory stimuli (1–5). Anatomically, neuroimmune interfaces have been discovered and studied within the spleen (6, 7), intestines (8, 9), the adrenal gland [HPA/cortisol (10) and dopamine reflex (11)], lymph nodes (12, 13), bone marrow (14), spinal column (15), pancreas (16), and heart/cardiovascular system (17). Both vagal and sympathetic afferent nerves have been shown essential in sensing and communicating peripheral immune status to the brain, which then modulates outflow to effector/immune cells within these interfaces to maintain homeostasis (18). Of these, the splenic neuroimmune interface has been intensively studied (1, 6, 7, 18, 19). Implant-based or pharmacological stimulation of this reflex, named the splenic cholinergic anti-inflammatory pathway (CAP) (3, 6, 7, 20, 21), has been shown to mediate control of specific cytokine production [including tumor necrosis factor (TNF)]. Mechanistically, it has been shown that vagus nerve stimulation results in norepinephrine (NE) release within the spleen (19). Splenic T cells are then modulated by the NE, and release acetylcholine (ACH) (1, 6, 7). This increase in splenic ACH results in inhibition of macrophage TNF production through alpha7 nicotinic acetylcholine receptor signaling (7). More recently, this anti-inflammatory pathway has been shown to be controlled by a cluster of cholinergic neurons within the dorsal motor nucleus (DMN), which projects to the celiac ganglion, and when activated results in the increased splenic nerve activity associated with cytokine/TNF inhibition (18). On the afferent side of the pathway, sensory neurons within the nodose ganglion (expressing specific cytokine receptors) have been shown to communicate cytokine specific nerve signals to the central nervous system (22, 23) and are hypothesized to modulate the activity (and thus level of cytokine suppression) of CAP. In addition, pain is likely sensed by nociceptors in infected tissue (such as lungs) and may also contribute to sensory modulation of neuroimmune pathways (24–26). Thus, significant evidence shows that these pathways contain the neuro-immune components to both sense inflammatory status (through afferent neurons) and respond to modulate cytokine production. Despite the extensive mechanistic investigation of the components of the splenic CAP, models used to study the effect of CAP activation have typically utilized systemic [i.e., severe endotoxemia (19)] or pathological [i.e., models of disease, such as collagen-induced arthritis (27)] inflammatory stressors. Therefore, the status of CAP activity during, and the contribution of the neuroimmune pathway response to, local/acute inflammatory stressors and infection remain unknown.

Several practical challenges have impeded the study of CAP stimulation during the progression of, and response to, local/acute infection. First, studies using implanted nerve stimulators [such as VNS (6, 18, 19)] are invasive and remain practically challenging, requiring surgical implantation of the device and long healing times to ensure that the inflammatory effects of the surgery do not impede the study. Second, use of electrical implants or pharmaceutical neuroimmune stimulators provide imprecise methods of neuroimmune modulation. For instance, cervical VNS implantation places the stimulator on a major vagus nerve trunk, potentially causing activation of many underlying neuroimmune (and other) reflexes during stimulation (3, 28, 29). Smaller electrical stimulators may enable near-organ implantation, and more precise activation of neurons entering specific organs (30, 31). However, low-invasive methods for laparoscopic implantation of these miniature stimulators are still under-development, and not available to the broader scientific community. In addition to implants, several pharmacological agonists of molecular components of neuroimmune pathways have also been discovered (32, 33). However, typical intravenous (i.v.) methods of administration often results in non-specific modulation of neuroimmune interfaces across the body (e.g., inhibition of MAPK signaling in both splenic and intestinal CAP locations using i.v. administration of semapimod), and/or require invasive methods of administration to modulate a specific neuroimmune interface (such as intracerebroventricular (i.c.v.) administration of semapimod).

Recently, our group (12, 28, 29, 34–38) and others (39–42) have shown the capability of pulsed ultrasound stimulation [targeted to either near organ (36, 43), whole organ (39, 40), or sub-organ anatomy (28)] to precisely modulate underlying nerve reflexes. In the spleen, the Okusa Lab (40–42) has shown that whole spleen ultrasound stimulation prevents ischemia-reperfusion injury (IRI) *via* the splenic CAP. Pathway specificity for splenic CAP (versus intestinal CAP or other neuroimmune pathways) was shown, as both physical splenectomy and chemical sympathectomy (with 6-OHDA) prior to ultrasound eliminated the protective effect. Furthermore, adoptive transfer of splenocytes from ultrasound-treated (but not sham) mice to naive mice was sufficient to protect recipients from IRI. In our group (28), splenic ultrasound reduced LPS-induced cytokine (e.g., TNF) release, and this protection was coincident with ultrasound-induced local but not systemic/plasma increases in CAP-related neurotransmitters. In addition, the ultrasound effect was not apparent in nude (lacking functional T cells), CD4 ChAT knock-out, or α7nAChR knock-out mice (key components of the CAP pathway). Splenic ultrasound was shown to have an equivalent effect on LPS-induced cytokine reduction but lack several of the associated off-target side-effects of cervical VNS, including effects on heart rate and metabolic function. Based on these previous findings, ultrasound-based CAP activation provides a new tool with which to study the effects of CAP stimulation over extended periods of time (i.e., days-weeks) non-invasively.

Many of the immunological steps associated with the progression of a local infection to a systemic and coordinated immune response are well-known. An initial innate immune response to infection includes upregulation of cytokines at the local site of infection, due to pathogen/inflammagen interactions or intracellular signaling with Toll-like and NOD-like receptors in immune cells, and activation of NF-κB-mediated (and other) intracellular signaling pathways (44). This local cytokine signaling then results in further leukocyte recruitment to the infected area. Upon elimination of the invading pathogen, these acute inflammatory responses become self-limiting, due to the release of resolvins and other anti-inflammatory molecules. However, failure to resolve the local infection or injury, dysregulation in the inflammatory response, and progression to unresolved and chronic inflammatory states can result in severe and lethal outcomes (including sepsis). It has previously been hypothesized that a physiological role of splenic CAP is to provide a protective mechanism that limits the potential negative effects of severe/systemic or chronic inflammation (1). This hypothesis has been tested by activating splenic or intestinal CAP prophylactically or immediately following severe infection or injury to provide protection prior to the challenge (1, 3, 6, 7, 18, 19, 40). These investigations have included models of endotoxemia (18, 21), sepsis (21), hemorrhagic shock (45), postoperative ileus (46), and kidney ischemia reperfusion injury (40). This hypothesis has also been tested in models of inflammatory disease [such as collagen- or serum-induced arthritis (27, 39) and chemical-induced colitis (35)], in which the CAP pathway is activated to provide an anti-inflammatory effect in the presence of chronic inflammatory disease. In each of these models, electrical (2), ultrasonic (28), or pharmaceutical (32) activation of CAP resulted in reduced cytokine production and disease-alleviating therapeutic effects.

However, there have been no studies investigating the effects of CAP activation during the progression and resolution of a localized and acute infection. Interestingly, it was recently shown that LPS challenge did not induce a TNF response in models of post-sepsis survival (i.e., animals surviving a septic episode that exhibit persistent immune impairment and immune fatigue, or compensatory anti-inflammatory response syndrome) (47). In these experiments, the TNF response to LPS could be rescued by first pharmacologically blocking the CAP pathway, demonstrating that suppression of the LPS-triggered TNF response in sepsis survivors was likely due to constitutive vagus nerve activation. We therefore hypothesized that the LPS response may also be suppressed during other physiological periods of natural CAP activation, such as the response to an acute infection prior to its resolution.

Herein, we applied a model of acute lung inflammation by first titrating the inoculum of *S. pneumoniae* during intra-tracheal instillation in rats (48), and then identifying the inoculum that resulted in positive bacterial lung cultures 16 hours following infection but did not result in systemic bacteremia (enabling symptom reduction (e.g., lower pain score) within 48 hours). We then investigated CAP status using the standard whole blood TNF response assay (7, 18, 20, 28) at multiple timepoints (i.e., 4-, 16-, and 48-hours) following infection. We measured a reduced cytokine response during infection, including complete suppression of the cytokine response at 16 hours (corresponding with the time of most severe symptoms and innate immune response markers). We next activated CAP using non-invasive ultrasound-based neuromodulation (28, 29, 34, 37, 39–42) and demonstrate that further stimulated CAP suppression of the TNF response was also time- or immune status-dependent during infection. That is, CAP stimulation did not result in further suppression of cytokine response during the intermediate phase (i.e., 16 hours following inoculation) of infection, but was observed immediately following instillation/challenge (i.e., hours) and returned within 48 hours. We also performed plasma cytokine and blood cell transcriptomic profiling and demonstrate that the 16-hour timepoint (in which CAP-mediated cytokine suppression was not observed) was associated with a peak in plasma CXCL1/KO response (neutrophil chemokine) and a transcriptional-shift in circulating immune cells.

## Materials and Methods

### Animal Model

Adult male Sprague-Dawley rats (age 9-11 weeks) weighing 300-400 gm were used in this study under the approval of Institutional Animal Care and Use Committee (IACUC) at the Feinstein Institutes for Medical Research. Rats were housed in 12 hours light/dark cycle with continuous access to rat chow and water. Animals were allowed to acclimate for 5 days before including them in the study.

### Bacteria Preparation and Lung Instillation

Streptococcus Pneumonia Serotype 19F [49619, American Type Culture Collection (ATCC) was used in this study. The bacteria were cultured in Brain Heart Infusion (BHI) broth until it reached the log phase with an optic density (OD) of 0.5 at 600 nm. It was then aliquoted in 1.8 ml cryogenic vials with 30% glycerol and stored in a -80°C freezer. All experiments were performed using the same batch of stock bacteria to avoid variability between experiments. A day before the experiment, a vial was taken out of the freezer, and 200 ul was inoculated in 500 ml sterilized BHI broth. The broth was left overnight in the incubator at 37°C and 5% CO_2_, until an OD_600_ of 0.5 was reached. A growth curve was generated to determine the amount of bacteria present at specified optical densities. Calculations were made to determine a specified dose of bacteria to be delivered. The broth was centrifuged, and the pellet was resuspended in sterile phosphate buffer saline. The inoculum was kept at 4°C until immediately prior to instillation.

Lung instillation was performed inside the Biosafety Hood (BSL-2). Rats were anesthetized with isoflurane (5% for induction and 2-3% for maintenance). Body temperature was maintained at 37°C using a heating pad connected to the water circulating pump. Once the rats were under anesthesia, they were placed in a supine position, and hairs were removed using clippers and depilatory cream. The neck area was then cleaned using an aseptic technique, as described before (49). 1-2 cm horizontal skin incision was given on the neck, and salivary glands were separated. The sternohyoid muscle was retracted to visualize the trachea and larynx. A 20G catheter was placed in the trachea below the larynx (IV Catheter 20G x 1-3/4in L, Jelco). A polyethylene tube (PE-10) was then inserted inside the catheter, and a bacterial dose of 3 x 10^5^ CFU suspended in 200 μl saline was instilled. The dose was confirmed in every experiment by culturing the calculated suspension on sheep blood agar plates. The catheter was removed immediately after the inoculation, and rats were kept in an upright position for 20 seconds to make sure the inoculum had reached the lungs. The skin was then sutured using 4.0 Nylon suture, and the rats were allowed to recover under the heat lamp.

### Splenic Ultrasound Neuromodulation

Splenic ultrasound stimulation (28) was delivered at 4-, 16-, or 48-hours after bacteria or saline inoculation. The rats were anesthetized using isoflurane and placed in a right lateral decubitus position. The hairs were removed from the left flank area using clippers and depilatory cream. The location of the spleen was identified using diagnostic imaging ultrasound and marked with a pointer, as used in a previous study (28). The ultrasound gel was placed at the marked area which was followed by the ultrasound probe. The stimulation was then turned on for 5 minutes. The rats were then allowed to recover from anesthesia under the heat lamp. The research ultrasound system was utilized to deliver stimulation, as described in detail previously (28). Briefly, the system consists of RF power amplifier (E&I 350L), a function generator (Agilent 33120A), a matching network (Sonic Concept), and High-Frequency Focused Ultrasound Probe/transducer (Sonic Concept H106). The stimulation parameters used were sine waves with a frequency of 1.1 MHz, amplitude of 200 mVpp, burst cycles of 150, and a burst period of 200 ms.

### Physiological Measurements and Grimace Score

In some experiments, the electrocardiography (ECG) was recorded using a 3-leads ECG on the limbs, and body temperature was recorded using a rectal temperature probe. These signals were recorded using a data acquisition system PowerLab (ADInstruments) and visualized in the LabChart software (ADInstruments). The heart rate was calculated from the ECG.

**Figure 1:**
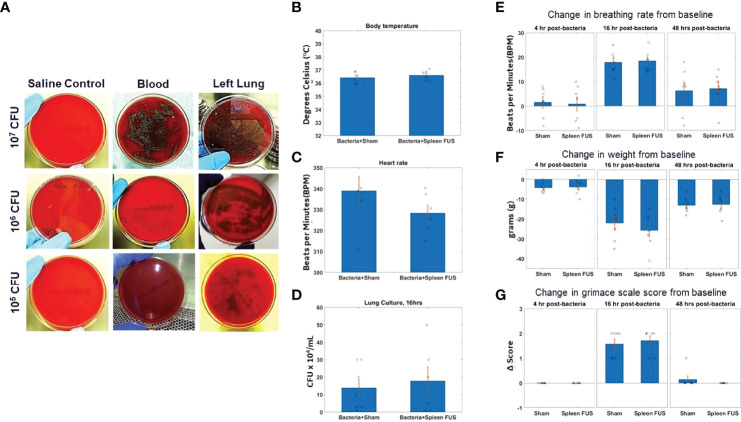
S. Pneumonia dose titration and change in physiological markers. **(A)** Blood and lung cultures in sheep blood agar plates at 16 hours timepoint after bacterial inoculation of different doses from 10^5 to 10^7 CFU. The left column shows lung culture plates after saline instillation. The middle and right columns shows blood and lung culture plates after bacterial inoculation as different doses. (**B–D**; n=6) Body temperature, heart rate and lung culture after 16 hours of bacterial inoculation in sham and splenic ultrasound groups. (**E, F**; n=7) Change in breathing rate and body weight from the baseline (i.e., before bacterial inoculation) at 4 hours, 16 hours, and 48 hours post bacterial infection/instillation. **(G)** Grimace score for pain assessment at 4 hours, 16 hours, and 48 hours post bacterial infection/instillation. Grimace score reflects 0=normal, 1=moderate, and 2=obvious.

The grimace scale was used to assess the level of pain in the animals. Measures were made by unblinded observer at 4-, 16-, or 48-hours timepoints post-inoculation. Four animal response/anatomical regions of the animals were observed, namely, orbital tightening, nose/cheek flattening, ear changes, and whisker changes. Each component was given a score of 0=normal, 1=moderate, and 2=obvious. We monitored each of these 4 components, and a toal score was given for each on the scale 0-2; the total socre was used in **Figure 1G**.

### Blood and Tissue Samples

Pre- and post-ultrasound or sham blood samples were collected in EDTA-coated tubes to prevent coagulation. Blood for the first sample was collected from the tail vein, and the second sample was from cardiac puncture. The blood was either used for plasma collection, whole blood cytokine response assay, peripheral blood mononuclear cell (PBMC) response assay, complete blood count analysis, or RNA sequencing. For plasma collection, blood samples were centrifuged at 2000 RCF for 10 minutes. The supernatant was collected and stored in a -80°C freezer. Lungs were collected in some experiments to confirm a bacterial infection. Right and left lungs were separately placed in 1 ml of PBS. Lungs were homogenized and serially diluted in PBS. The dilutions were then plated in sheep blood agar plates and placed in the incubator at 37°C. Colony-forming units (CFU) were counted the following day. In a few experiments, spleens were collected and stored at -80°C, which were later used to measure splenic neurotransmitters.

### Whole Blood and PBMC Cytokine Response Assay

The whole blood or PBMC response assay was performed in both pre- and post- ultrasound samples. Blood was collected in EDTA-coated tubes to prevent coagulation. For whole blood cytokine response assay, 500 μl of blood was placed in an Eppendorf tube. The endotoxin Lipopolysaccharide (LPS) dose of 10 ng/ml was used to stimulate blood for the LPS-challenge test (L2630, LPS from Escherichia Coli O111:B4, Sigma Aldrich). The tubes were placed on a rocker inside the incubator at 37°C for 4 hours. The samples were then taken out of the incubator and centrifuged at 6000 RPM for 5 minutes. The supernatant was taken and stored in -80°c freezer until further analysis. All samples were run in duplicates.

For peripheral blood mononuclear cells (PBMC) cytokine response assay, 1 ml of blood was collected in EDTA-coated tubes and centrifuged at 1000 RCF for 15 minutes. In the meantime, 4.5 ml of a density gradient medium (Lymphoprep, Stemcell Technologies) was placed in a SepMate-15 tube (Stemcell Technologies). Once the blood samples were centrifuged, supernatant was taken out and the remaining cells at the bottom of the tube (~0.5 ml) was mixed with equal amount of PBS with 2% fetal bovine serum (FBS). The mixture was then carefully placed into the SepMate-15 tube using pipette. The SepMate-15 tube was centrifuged at 1200 RCF for 10 minutes at room temperature. After centrifuging, the top layer (cloudy) containing mononuclear cells (MNCs) was pipetted out and placed in a new falcon tube. The tube was centrifuged again at 300 RCF for 8 minutes to pellet. After centrifuging, the supernatant was vacuum out without removing pellet at the very bottom of the tube. Enriched MNCs were washed by resuspending the pellet with 1 ml PBS with 2% FBS. 10 μl of this sample was then taken and mixed with 10 μl of Typhan Blue Stain to count the number of MNCs/ml. Cell count was performed using a Countess-2 Automated Cell Counter (ThermoFisher). After cell count, the tube was centrifuged at 300 RCF for 8 minutes, and supernatant was vacuumed out without removing the MNCs pellet at the bottom, which was resuspended by adding a media (containing RPMI, 10% FBS, and Penn/Strep/Glutamine 2%). The volume of the media was calculated to make sure the final cell count of 500,000 cells/100 μl. 100 μl of this suspension was plated in a 96-well flat-bottom plate. An additional 100 μl of media was added to the well, which contained endotoxin LPS (L2630, Escherichia Coli O111:B4, Sigma Aldrich), making the final volume of 200 μl. A dose of 10 ng/ml was used to stimulate MNCs. The 96-well plate was then placed in the incubator at 37°CC for 3 hours. After incubation, the supernatant was taken and stored in a -80°CC freezer until further analysis.

### Cytokine Analysis

Plasma, whole blood assay, and PBMC samples were analyzed using a V-Plex Proinflammatory Panel 2 Rat Kit (Meso Scale Diagnostics). This kit can detect nine cytokines, namely TNF-α, IL-4, IL-5, IL-6, IL-10, IL-13. IFN-γ, IL-1β, and KC/GRO. All samples were run in duplicates as recommended by the manufacturer. Analyses were done using MSD Discovery Workbench analysis software (Meso Scale Diagnostics).

### Blood Cell Transcriptomics

RNA sequencing was performed in the 16-hour timepoint post-ultrasound or sham blood samples only. RNA extraction from tissues and RNA sequencing were performed at the Feinstein Institutes for Medical Research (FIMR), Genomics Shared Resource, Manhassett, New York. The quality was assessed by the RNA integrity number (RIN) from a BioAnalyzer (Agilent Technologies; **Supplementary Table 1**). Sequencing RNA libraries were prepared using the TruSeq Stranded mRNA Sample Preparation Kit (Illumina, San Diego, CA) according to the manufacturer’s instructions. Sequencing was performed on the NextSeq 550 Sequencing platform that output 75-base pair pair-ended reads at >30 million reads per sample. The RNA-seq data was processed at GE Global Research using established bioinformatics software tools. Base quality control was checked and found to be excellent using Fast QC v0.10.1 from Babraham Bioinformatics. Sequencing reads were mapped to the annotated rat genome version, Rattus norvegicus Rnor 6.0.91, using STAR_2.5.3a aligner. Transcript abundance estimates were then generated using RSEM which outputs the expected count for each transcript.

Transcript count normalization and differential expression analysis was performed on all samples using the DESeq2 tool. The p-values attained by the Wald test are corrected for multiple testing using the Benjamini and Hochberg method. Transcripts with an adjusted p value < 0.1 were counted as being differentially expressed. Output from DESeq2 included the median ratio normalization (MRN) values for each transcript of each sample. These normalized values were used for gene set enrichment and FARDEEP cell fraction analysis.

Gene set enrichment analysis (GSEA) was performed using GSEA (version 3.0) tool to identify functional pathways with the gene set collection Gene Ontology (GO) biological processes (C5), Reactome and KEGG curated genes sets (C2) and the hallmark gene sets (H) available at the Molecular Signatures Database (MSigDB). For GSEA, the DESeq2 MRN values were inputted into the GSEA tool for each gene in which its transcript had a log2 fold change with a p-value < 0.2. Gene sets identified by the GSEA tool to have a Familywise-error rate (FWER) p-value < 0.1 were considered significant. The FWER was used over the alternatively provided FDR statistics to minimize false positive findings.

Deconvolution of the cell fractions contained in the blood samples estimated from transcriptomics was computed using the FARDEEP algorithm that is implemented as a R package (50). The bulk RNA-seq data was deconvoluted using the molecular signature datasets LM22 and TIL10. The LM22 signature contains 22 immune cell types and was developed from Affymetrix Microarray data (51). The TIL10 signature contains 10 immune cell types developed from RNA-seq data (52) and was downloaded from Bioconductor as part of the quantiseqr package (quantiseqr: Quantification of the Tumor Immune contexture from RNA-seq data. R package version 1.2.0.).

### Splenic Neurotransmitter Measurements

Spleens were taken out from -80°C freezer and homogenized with 0.1-M perchloric acid, as described previously in detail (28). Briefly, the homogenate was centrifuged for 15 minutes, and supernatant was taken, which was injected into High Performance Liquid Chromatography (HPLC). HPLC with inline ultraviolet detector was used to analyze norepinephrine and epinephrine. The UV detector was kept at 254 nm wavelength, known to capture the absorption for norepinephrine, epinephrine, and dopamine.

### Hematology and Blood Count Measurements

In some experiments, a complete blood count (CBC) was performed specifically to measure white blood cells. Both pre- and post-ultrasound/sham samples were analyzed using ADVIA 2120i Hematology System (Siemens). The CBC analysis machine was routinely calibrated using normal blood, low-reticulocytes, and high-reticulocytes samples provided by Siemens as control.

### Statistical Tests

The statistical tests performed throughout were non-parametric Wilcoxon signed-rank test, unless otherwise stated. Mann-Whitney U-test was used to run statistics on receiver operating curves. The results were deemed statistically significant if p<0.05.

## Results

### Intratracheal Instillation and *S. pneumoniae* Challenge Dose Titration

To study the effects of cholinergic anti-inflammatory pathway activation (2, 28, 40–42) in an acute lung infection model (48), we first titrated the challenge dose of *S. pneumonae* in intratracheal instillations (**Supplementary Figure 1**) in rats. **Figure 1A** shows images of bacterial cultures taken from blood and lung tissue for bacteria instillations challenges from 10^5 – 10^7 CFUs (sampled at 16 -hour post-challenge timepoint). Challenges that contained greater than 10^5 CFU bacteria were found to result in septicemia, as measured by positive blood cultures. However, bacterial instillations containing 10^5 CFU bacteria remained within the lung tissue and did not result in positive blood cultures (n=7). **Figures 1B, C** shows that body temperature and heart rate were within the normal range for animals challenged with 10^5 bacteria at the 16-hour timepoint, and that splenic ultrasound stimulation did not change either parameter. Viable CFUs were cultured from the collected lung samples at 16 hours, demonstrating active infection at this timepoint, and again the total CFUs in the sham versus splenic ultrasound stimulated animals were not statistically different (**Figure 1D**). For the maximum level of challenge bacteria that did not result in septicemia (i.e., 10^5 CFU instillation), the maximum change in breathing rate, weight loss, and grimace score (pain assessment) was measured at 16 hours post-instillation, and these measurements again did not differ between sham and splenic ultrasound stimulation animals. The change in breathing rate (**Figure 1E**), weight loss (**Figure 1F**), and pain (**Figure 1G**) returned toward baseline levels by the 48 hours timepoint, suggesting an effective immune response.

### Whole Blood Cytokine Response Is Suppressed Following Bacterial Challenge or Ultrasound-Based CAP Activation

We next measured whole blood TNF after *ex vivo* LPS challenge, using blood samples collected at several time-points following the bacterial challenge (10^5 CFU challenge dose). We also performed the experiments in non-challenged control animals. **Supplementary Figure 2** (S2A; no challenge, time 0) shows that levels of TNF suppression due to splenic ultrasound stimulation and CAP activation (28, 40–42) was equivalent to previously reported data. The splenic ultrasound stimulus utilized herein has been previously shown to provide optimal CAP activation (i.e., complete TNF suppression to pre-challenge levels in an LPS-induced inflammation model) (28).

**Figure 2:**
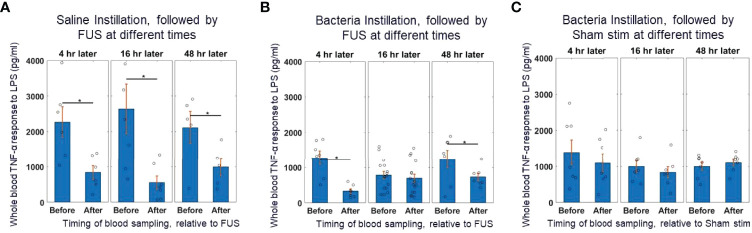
Whole blood cytokine response assay to saline and bacteria instillation. **(A)** TNF-α response to *in-vitro* LPS challenge test (10 ng/ml) in instillation naïve animals (left bars) and saline instilled animals (middle and right bars) before and after splenic ultrasound stimulation at multiple timepoints. **(B)** TNF-α response to *in-vitro* LPS challenge test in bacteria instilled animals before and after splenic ultrasound stimulation at 4 hours, 16 hours, and 48 hours post-bacterial infection. **(C)** Same as **(B)**, but in sham animals (i.e, without active ultrasound stimulation). Asterisk indicates *p*<0.05 using non-paramteric Wilcoxon rank sum test. n=7 for all groups (except bacteria instillation + CAP activation which has n=13).

**Figure 2A** shows that in animals that received saline instillation, whole blood TNF levels before FUS were relatively stable at 4, 16 and 48 hours after the instillation. After those animals received spleen FUS, TNF levels were suppressed at all time points. In animals that received bacteria instillation (**Figure 2B**), whole blood TNF was reduced by almost 50% (compared to saline instillation), at all time points; the suppressive response to FUS was maintained at 4 hours post-instillation, was absent at 16 hours post-instillation, and had returned at 48 hours post-instillation, indicating a time-dependent effect. As expected, animals that received bacteria and sham stimulation (**Figure 2C**) had reduced TNF levels before sham stimulation and there was no suppressive effect of stimulation.

This data shows that reduction in cytokine response due to the ultrasound-based CAP activation (in control animals that did not receive the bacterial challenge) was equivalent to the suppression response observed at the 16-hour timepoint in bacteria challenged animals. This suggests that during the bacterial challenge the TNF response is already maximally suppressed before any further CAP activation, such as post-infection application of the ultrasound stimulus. This data supports the previous hypothesis that CAP is activated during acute infections (1), is modulated based on the level of inflammation (19, 22, 23), and is maximally active during periods of strong innate immune response and inflammation (53).

### Plasma Cytokine/Chemokine and Blood Cell Profiling in Ultrasound Stimulated vs. Non-Stimulated and Control Animals

**Figure 3:**
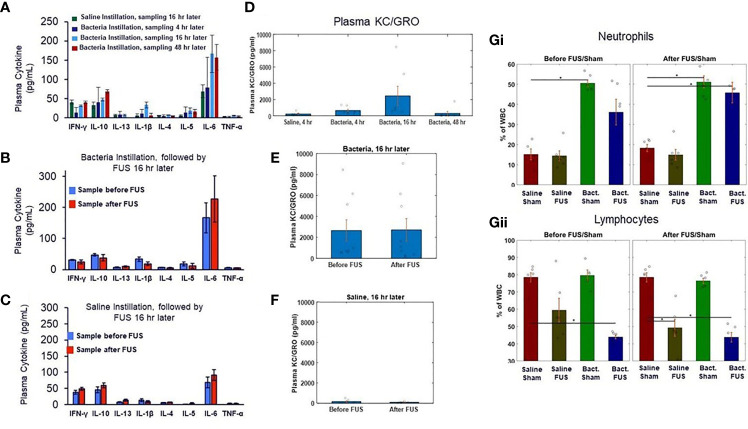
Plasma cytokine levels and blood cell counts after saline and bacterial instillation. **(A)** Plasma cytokines levels in pre- ultrasound/sham samples in control and bacteria instilled animals at 4 hours, 16 hours, and 48 hours timepoints. **(B)** Plasma cytokines levels in pre- and post- ultrasound samples in bacteria instilled animals at 16 hours timepoint. **(C)** Same as **(B)**, but in saline instilled animals. **(D)** Plasma chemokine KC/GRO levels (a neutrophil chemokine) in control (no bacteria) and bacteria instilled animals at 4 hours, 16 hours, and 48 hours timepoints. **(E)** Plasma chemokine KC/GRO levels in bacteria instilled animals before and after ultrasound stimulation at 16 hours timepoint. **(F)** Same as **(E)**, but in saline instilled animals. **(Gi)** Percentage of white blood cell differentials in saline and bacteria instilled animals before ultrasound or sham stimulation at 16 hours. **(Gii)** Same as **(Gi)**, but after ultrasound or sham stimulation. Asterisk indicates *p*<0.05 using non-paramteric Wilcoxon rank sum test.

We further examined additional plasma samples taken at the same timepoints as the whole blood TNF response assays shown in **Figure 2**. The localized lung challenge did not result in significant changes to most circulating cytokines (**Figure 3A**), compared to the non-infected controls. Furthermore, ultrasound stimulation did not result in further reduction of these circulating cytokines from this low baseline level in ultrasound **(** **Figure 3B** **)** or sham-treated cohorts **(** **Figure 3C** **)**. Methods of presymptomatic or asymptomatic identification of infection in the absence of detectable changes in systemic markers, such as cytokines, is an active area of research and desirable to aid in infectious disease monitoring and control (54, 55). Circulating concentration of the chemokine KC/GRO (i.e., rodent equivalent of CXCL1; a neutrophil chemokine) was elevated 4-hours post-infection, further elevated at 16-hours, and returned towards baseline (i.e., levels measured in the no bacteria controls) at 48 hours following infection **(** **Figure 3D** **)**. This chemokine is known to induce neutrophil influx into lung tissue and is required for lung clearance following a bacterial infection (56). Although elevated in the bacteria challenged animals, this chemokine was not altered by ultrasound treatment in either bacteria **(** **Figure 3E** **)** or saline groups **(** **Figure 3F** **)**. Complete blood analysis (**Supplementary Figure 3**) and blood cell differentials (**Figure 3G** and **Supplementary Figure 3**) were then performed to test if bacterial-induced changes to the chemokine (or other) profile resulted in gross changes in circulating blood counts. As expected, the total number and percentage of neutrophils (compared to leukocytes) was increased 16 hours after infection (compared to non-infected controls). However, none of the gross blood parameters measured were significantly different in the post-ultrasound or post-sham treated (compared to pre-ultrasound) samples.

Additional splenic sample measurements also revealed no significant difference in CAP-related neurotransmitter concentrations between the groups at the 16-hour timepoint (taken 2 hours after ultrasound or sham stimulation; **Supplementary Figure 4**). Compared to previous reports, the concentration of splenic norepinephrine and acetylcholine (the primary CAP signaling components) were comparable to the naïve and fully-activated/US stimulated groups (i.e., compared to the LPS-challenged group in which CAP signaling was suppressed (28)). This data again supports active CAP signaling in both the ultrasound-stimulated and bacteria-challenged groups, herein.

### Blood Cell Transcriptomic Profiling in Ultrasound Stimulated vs. Non-Stimulated and Control Animals

At the 16-hour time-point 4,985 genes were differentially expressed in blood samples taken from the saline versus bacteria injected groups (**Supplementary Figures 5A, B**). In contrast, only 4 genes were differentially expressed (adjusted p-value < 0.1) between the ultrasound stimulated and ultrasound sham groups (**Supplementary Figure 5B** **).** Further sub-group analysis (**Supplementary Figures 5C, D**) showed that compared to the ultrasound sham group, the ultrasound stimulation group exhibited ~8% less differentially regulated genes between the bacteria and saline instillation groups (i.e., decrease from 3037 to 2780 differentially expressed genes between bacteria and saline installation groups for the ultrasound stimulated and ultrasound sham groups respectively). In contrast, differential regulation observed across the saline groups (i.e., 222 differentially expressed genes between ultrasound stimulated and ultrasound sham) was almost completely abolished by bacteria instillation (i.e., 6 differentially expressed genes between ultrasound stimulated and ultrasound sham).

Further gene set enrichment analysis (**Supplementary Figure 6A–F**) confirmed that the differentially expressed genes between bacteria versus saline instilled groups were specific to an acute infection indicated by the top five gene sets (i.e., regulation of inflammatory response, inflammatory response, innate immune response, response to bacterium, and response to type I interferon). Thus, at the16 hour timepoint (during the peak of the innate immune response to the infection) the whole blood RNA sequencing signatures are dominated by the response to bacteria compared to the effect of ultrasound CAP stimulation. However, the effect of CAP (on cytokine response) has been shown to be primarily mediated through monocytes/macrophages, an effect that may be masked when examining whole blood gene expression changes across all blood cell types.

We therefore next examined the cell fractions present in the pre- and post-ultrasound (or sham) samples by deconvolving the bulk RNA sequencing data using the FARDEEP algorithm and immune cell RNAseq signatures (**Supplementary Figure 7**–**9**). In the saline instilled groups, ultrasound stimulation had the most significant impact on genes within the TIL10 Macrophages M2 gene signature (**Supplementary Figures S7** and **S9**). The fraction of these genes, in both bacteria-instilled groups (ultrasound stimulated and ultrasound sham) were lower compared to the saline-instilled ultrasound sham group (p-value < 0.005), but not statistically different to the saline-instilled ultrasound stimulated group (**Supplementary Figure 9A**). Further analysis revealed that 13 of the top 20 differentially expressed genes within this Macrophage M2 signature have been previously associated with dendritic cell maturation and monocyte polarization (**Supplementary Figures S8B, C**). This differential expression in Dendritic Cell gene signatures between bacteria-instilled versus saline-instilled groups was also apparent in the LM22 total dendritic cell (p-value < 0.0069) and resting dendritic cell (p-value < 0.039; **Supplementary Figure 8**) signatures. These findings further support the cytokine response (**Figure 2**) data above, which showed that at the 16-hour post-instillation timepoint the cytokine response (i.e., CAP activity) was already maximally suppressed, and additional ultrasound stimulation had no effect on cytokine output.

### Ultrasound Stimulation of the Spleen Improves the Sensitivity and Specificity in the Diagnosis of Lung Infection

**Figure 4:**
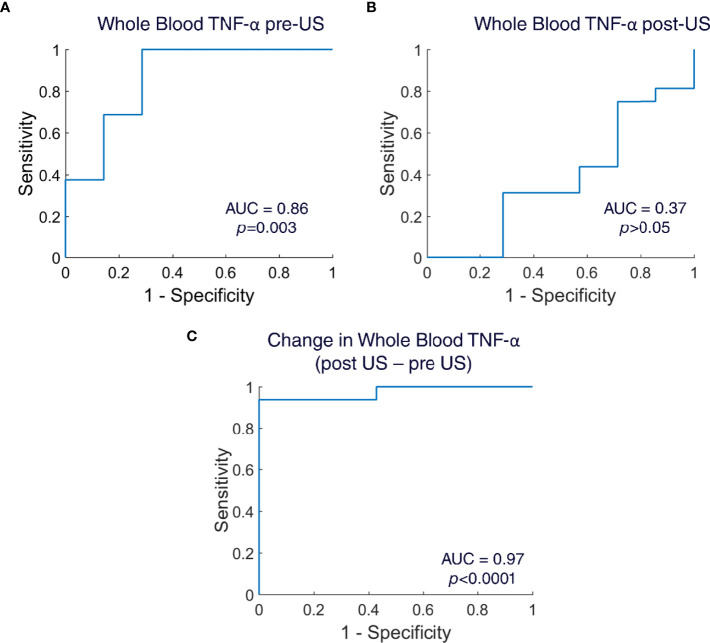
Receiver operating curves (ROC) for whole blood TNF-α. **(A)** ROC created for whole blood TNF-α pre-US samples (n=23 animals; Area Under the Curve=0.86; Mann Whitney U-test, p=0.003). **(B)** ROC created for whole blood TNF-α post-US samples (n=23 animals; Area Under the Curve=0.37; Mann Whitney U-test, p>0.05). **(C)** ROC created for change in whole blood TNF-α (post US – pre US) (n=23 animals; Area Under the Curve=0.97; Mann Whitney U-test, p<0.0001).

To assess if the ultrasound stimulation (US) adds any value in the diagnosis of lung infection, we used the TNF-a values from the whole blood LPS assay from both disease and healthy animals. We calculated sensitivity and specificity and created Receiver Operating Curves (ROC) for the whole blood TNF-a pre-US, for the whole blood TNF-a post-US, and for the change in whole blood TNF-a (post US – pre US). The diagnostic accuracy was determined by the calculating Area Under the Curve (AUC). The AUC in the whole blood TNF-a pre-US was 0.86 (Mann-Whitney U-test, p=0.003), which indicates that the whole blood LPS assay pre-US itself without any ultrasound intervention has high sensitivity and specificity for the diagnosis of infection (**Figure 4A**). The AUC in the whole blood TNF-a post-US was 0.37 (Mann-Whitney U-test, p>0.05), which indicates poor diagnostic accuracy (**Figure 4B**). Interestingly, when the AUC was calculated for the change in whole blood TNF-a (post US – pre-US), the diagnostic accuracy of the test increased even higher than the whole blood TNF-a pre-US alone (AUC: 0.97; Mann-Whitney U-test, p<0.0001) (**Figure 4C**). These AUC values indicate that the ultrasound stimulation of the spleen is a promising diagnostic test that has the capability to detect infections.

## Discussion

It has been hypothesized that the physiological role of the cholinergic anti-inflammatory pathway (CAP) is to limit excessive systemic activation of the immune system, during response to infection (1, 3, 53). Evidence suggests that the nervous system is capable of sensing peripheral inflammation (through afferent neurons) and providing an integrated response to dampening the immune system (through efferent neurons) during periods of excessive inflammation (1, 5, 19, 22, 23). This protective effect has been shown by activating CAP prophylactically or immediately following injury in multiple models of severe inflammation or trauma, including sepsis, haemorrhagic shock, postoperative ileus, and kidney ischemia reperfusion injury (1, 6, 19, 21, 40, 45, 46). The protective effect has also been shown in models of chronic and pathological inflammation, including collagen-induced and serum transfer models of arthritis and DSS-induced colitis (20, 27, 35, 39). However, the activity of CAP, and the effect of stimulating the CAP pathway during progression of a local or acute infection has not previously been measured.

Herein, we utilized the whole blood cytokine response assay to monitor CAP activity [i.e., the cytokine response (20, 28)] at various timepoints following infection, and measured the effect of additional CAP stimulation (i.e., splenic ultrasound-based activation) at those same post-infection timepoints. The bacterial challenge was first demonstrated to result in positive lung culture (i.e., infection) 16 hours following infection, but did not result in systemic bacteremia, and enabled reduction in symptom measures (e.g., pain) within 48 hours. Weight loss was observed in the model, despite lack of bacteremia. However, weight loss during lung infection has been previously shown to correlate with cytokine concentrations in bronchial lavage (not systemic blood measures) (57). In addition, several lung specific mechanisms responsible for weight loss during lung infection are under-investigation including the effects of IL-22 upregulation in infected lungs (58), alterations in gut microbiota post-infection (59), and decreased food intake (57). Our results showed complete cytokine suppression in the whole blood response assay (i.e, maximum CAP activation) at 16 hours post-infection without any additional ultrasound-based CAP stimulation, which recovered to ~50% cytokine suppression (compared to non-infected controls) by the 48-hour timepoint. In non-infected animals, cytokine response was suppressed to the same level using the ultrasound stimulation procedure [previously shown to provide maximum CAP activation or full cytokine suppression in an LPS-induced inflammation model (28)]. Therefore, no additional cytokine suppression was observed after ultrasound-induced CAP activation 16-hours post infection (i.e., in animals already responding to the bacteria challenge and experiencing endogenous bacteria-induced CAP cytokine suppression).

The absence of cytokine suppression following exogenous CAP stimulation/activation has been reported in only one other previous report (47), that is, in animals surviving cecal ligation and puncture (CLP) induced polymicrobial sepsis. This previous report demonstrated that additional cytokine suppression following exogenous CAP stimulation/activation was not observed in the sepsis survivors due to constitutive endogenous activation of the CAP. That is, CAP was found to be pathologically active in the sepsis survivors (i.e., a potential mechanism associated with the prolonged post-sepsis state of immunosuppression and compensatory anti-inflammatory response syndrome). However, whether or not CAP is activated during a normal response to infection (i.e., an infection that resolves and does not result in systemic bacteremia or sepsis) has not been previously investigated. Herein, we demonstrate that cytokine response was suppressed during the progression of immune response to local/lung infection from initial challenge toward resolution. Furthermore, the level of suppression was time dependent (following the initial challenge) and reached a maximum level of suppression at the 16-hour post-challenge timepoint, which coincided with maximum bacteria-induced chemokine signaling and neutrophil mobilization.

Both blood cell counts (**Figure 3G**) and bulk RNA sequence profiling (**Supplementary Figures 5**–**8**) data confirmed a strong immune response to the infection at the 16-hour timepoint, including an increase in the circulating neutrophil/leukocyte ratio and upregulation of genes associated with inflammatory response to bacteria and its regulation. In addition, there was an observed effect of ultrasound stimulation on several monocyte polarization- and plasmacytoid/dendritic cell maturation-related gene signatures (60–67) (**Supplementary Figures S7**–**9**) in saline controls, which were all also observed in bacteria-instilled animals. This further validates the cytokine response data, which showed a maximal cytokine suppression (i.e., CAP activity) in bacteria challenged animals at 16 hours, and no additional effect of ultrasound stimulation at that time point. Future studies assessing gene expression at multiple time-points and measuring single cell/blood cell type specific gene expression during infection will be necessary to further examine the effect of CAP activity (both natural and stimulated) during the course of infection.

The whole blood cytokine response used herein, has become the standard assay for assessing CAP activation in both pre-clinical and clinical trials (19, 20, 28). However, we investigated CAP response in an acute infection model, and at multiple timepoints post-challenge, which have not previously been reported. These new experimental parameters led to the observation that CAP is activated during the normal course of immune response to a local/acute bacterial challenge, the level of activation is time dependent during the course of the immune response, and the maximal CAP activation coincides with the time period of maximal chemokine signaling and innate immune system mobilization. These observations are consistent with the long-standing hypothesis that the physiological role of CAP is to limit excessive and systemic activation of the innate immune system during an immune response (2, 53) but have not been previously measured. Despite this important initial observation in an acute infection model, several questions remain unresolved, and must be further addressed in future studies.

First, investigation of CAP activity across a larger range of challenge doses will be further informative. Based on the currently reported CAP hypothesis (i.e., that the efferent arm of CAP is modulated based on input signaling from cytokine and pathogen responsive afferents), the level and post-challenge timing of cytokine suppression/CAP activation should be expected to vary across different challenge doses. Furthermore, pharmacological blockade of CAP (28, 47) at pre- or post-challenge timepoints should modulate the pathogen effect on whole blood cytokine response. However, blocking of CAP may also affect the natural course of immune response and progression, and these experiments will require careful design (with respect to blockade versus challenge timing) to decouple those effects. The study herein focused on lung infection. However, afferent neurons project to many organs in the body, and the extent and time-course of CAP activation may be dependent on infection site location (22, 23). As an example, additional efferent arms of the anti-inflammatory pathway has recently been mapped within the intestinal tract (i.e., intestinal nerve pathways that modulate local macrophage activity and cytokine secretion independent of the splenic pathway) (8, 46). It is currently unknown if the site of local infection (and subsequent activation of different afferent and sensory neurons) results in differential activation of the splenic, intestinal, or other [i.e., the hypothalamic pituitary axis (HPA) or adrenal/dopamine] anti-inflammatory pathways. Simple observational studies, which measure the extent of activation of each of these different anti-inflammatory pathways by measuring their specific immunomodulatory effector [i.e., cortisol (HPA), dopamine (adrenal), TNF (intestinal vs. splenic/circulating)] under different inflammatory and immune challenges are warranted.

CAP activation [i.e., pharmacological (32), implant/electrical-based (20), or ultrasound-based (28, 39, 40)] has typically been studied in the context of testing a bioelectronic or neuromodulation-based therapy. For instance, implant/electrical-based CAP activation has been used to modulate cytokine levels in pre-clinical models of rheumatoid arthritis or irritable bowel disease (27, 68), and now in several human feasibility studies (20, 68, 69). In these studies, the underlying hypothesis is that CAP signaling is impaired in the disease and contributes to the elevated levels of cytokines (such as TNF) in the pathological state. In this respect, the mechanism associated with the CAP-based bioelectronic medicine (e.g., electrical/implant-based CAP activation, and cytokine/TNF reduction) is being investigated to replace a specific drug-based mechanism (i.e., cytokine TNF reduction) of pharmaceutical medications that are currently applied to the disease (i.e., anti-TNF based biologics). Based on the data we present herein, we speculate that the status of the CAP pathway (i.e., level of CAP activation) may also have further diagnostic relevance. We demonstrate that CAP is activated at different levels throughout the progression of the immune response to an infection, and that the whole blood cytokine response assay can be used to assess periods of CAP activity during this progression. Thus, the ability of the CAP stimulus to produce further cytokine suppression is indicative of the current immune state, and inflammatory response to the bacterial challenge. To further confirm the diagnostic ability of this test, we also calculated sensitivity and specificity, and created receiver operating curves (ROC). Indeed, the whole blood cytokine assay (pre-US sampling) has a capability to detect local infection with high sensitivity and specificity (**Figure 4A**). However, the diagnostic capability of the whole blood cytokine assay increased to near-ideal in the ROC of the change in whole blood cytokines (pre US – post US) (**Figure 4C**). This further indicates that ultrasound of the spleen adds additional value to the whole blood cytokine assay and can be utilized in the diagnosis of infections.

The whole blood assay has been the standard method of assessing CAP activity in past reports (1, 2, 7, 19, 20, 28). However, **Supplementary Figure 10** shows additional observations we have made by replacing whole blood with PBMCs during the cytokine response assay. Macrophages have been reported as the main immune cell components of CAP. However, both macrophages and neutrophils (which are removed during processing of whole blood into PBMCs) are known to be endogenous sources of TNF, and activated neutrophils are known to modulate macrophages toward an “M1” or proinflammatory phenotype (70). In the PBMC response assay (i.e., lacking granulocytes, such as neutrophils), the total TNF response (**Supplementary 10A**) was greatly reduced compared to the whole blood TNF response in the no bacteria controls (**Supplementary 2A**). In addition, there was no additional suppression in TNF response after splenic ultrasound stimulation in the PBMC samples. This suggests that neutrophils are required for eliciting a maximal cytokine response from macrophages within the response assays, and that observation of the canonical CAP effect requires this neutrophil-macrophage interaction. Furthermore, at the 16-hour timepoint ultrasound stimulation/CAP activation resulted in an increased TNF response in the PBMC samples. This again demonstrates a rapid differential effect of CAP pathway stimulation in challenged/infected versus non-infected cohorts, and further supports the need to study these neuroimmune pathways within the context of different immune and inflammatory states (including the initial hours or pre-symptomatic stages of infection).

In summary, we present the first data examining CAP activity (as measured using the standard whole blood cytokine response assay) during progression and resolution of a local/acute bacterial infection. Our data shows a time-dependent level of CAP activation during infection, including low-level activation at infection onset (i.e., hours after challenge), maximum activation at the 16-hour timepoint post-challenge, and a decrease in activation during progression toward infection/inflammation resolution. We demonstrate that the response to additional ultrasound-based CAP activation is also dependent on the time-frame post-infection that the animal is stimulated. We further speculate that this non-invasive ultrasound tool for CAP activation can be utilized to further investigate the diagnostic utility of the status of CAP activity during infection and infectious disease progression.

## Data Availability Statement

The original contributions presented in the study are publicly available. This data can be found here: https://www.ncbi.nlm.nih.gov/geo/query/acc.cgi?acc=GSE197466.

## Ethics Statement

The animal study was reviewed and approved by Institutional Animal Care and Use Committee (IACUC) at the Feinstein Institutes for Medical Research.

## Author Contributions

UA conceived and designed experiments, performed experiments, analyzed and interpreted experimental results, and wrote the manuscript. JG analyzed and interpreted experimental results. AD, OY, CM, and VC performed experiments, analyzed and interpreted experimental results. IM critically reviewed the manuscript. CD conceived and designed experiments, and reviewed the manuscript. SZ supervision, conceived and designed experiments, and analyzed and interpreted experimental results. CP supervision, conceived and designed experiments, analyzed and interpreted experimental results, and wrote the manuscript. All authors reviewed the manuscript. All authors contributed to the article and approved the submitted version.

## Funding

Funding for this work was provided by a BARDA contract GE 75A50119C00056. This project has been funded in whole or in part with Federal funds from the Department of Health and Human Services; Office of the Assistant Secretary for Preparedness and Response; Biomedical Advanced Research and Development Authority, DRIVe, under this contract. The funder was not involved in the study design, collection, analysis, interpretation of data, the writing of this article or the decision to submit it for publication.

## Conflict of Interest

Author CP, JG, VC, and CM is/was employed by General Electric. SZ and UA have received previous financial support from General Electric.

The remaining authors declare that the research was conducted in the absence of any commercial or financial relationships that could be construed as a potential conflict of interest.

## Publisher’s Note

All claims expressed in this article are solely those of the authors and do not necessarily represent those of their affiliated organizations, or those of the publisher, the editors and the reviewers. Any product that may be evaluated in this article, or claim that may be made by its manufacturer, is not guaranteed or endorsed by the publisher.

## Supplementary Material

Supplementary Table 1 Quality metrics for RNA sequencing of blood samples across all three experimental series. The whole blood samples were collected from all groups at 18 hours post bacteria/saline instillation and 2 hours post ultrasound stimulation/sham treatment. The samples were from three different experimental series performed weeks apart between each series. RNA quality and sequencing metrics were of high quality for each sequencing series, with no significant experimental batch effects. RNA integrity numbers (RIN) were >7 for each sample, total number of reads was >30 million for each sample, mean base quality was excellent > 27 (Phred), and percentage of reads mapped to a single location on the reference passed the requirement of > 50%.

Supplementary Figure 1 Dye controls used to test the intratracheal injection method. (left) Intratracheal tubes were inserted into both the right and left lung, and methylene blue dye was injected into each side. (right) An Intratracheal tube was inserted into the left lung only, and the methylene blue remained contained within the left lung.

Supplementary Figure 2 Whole blood Cytokine Response Plotted as Percent Maximal TNF/CAP Suppression. **(A)** TNF-α response to *in-vitro* LPS challenge test (10 ng/ml) in instillation naïve animals (left bars) and saline instilled animals (middle and right bars) before and after splenic ultrasound stimulation at multiple timepoints.). **(B)** % of TNF-α suppression in saline instilled animals. The total change in TNF-α response between pre- and post- ultrasound in naïve (no saline or bacteria instillation) animals (difference between left most blue versus red bars in 2A) was deemed as 100% TNF-α suppression. The pre- and post- ultrasound samples in saline instilled animals at different timepoints were compared with naïve TNF-α suppression and % of TNF-α suppression was calculated. **(C)** Same as **(B)**, but in bacteria instilled animals with ultrasound stimulation. **(D)** Same as **(B)**, but in bacteria instilled animals with sham stimulation. Asterisk indicates *p*<0.05 using non-parametric Wilcoxon rank sum test. n=7 for all groups (except bacteria instillation + CAP activation which has n=13).

Supplementary Figure 3 Complete blood count in all groups at the 16 hour timepoint. **(A)** Complete blood count analysis in saline and bacteria instilled animals with sham stimulation (no ultrasound) at 16 hours. WBC units are represented in 10^3 cells/μL; Red blood cell (RBC) in 10^6 cells/μL; Hemoglobin (HGB), mean corpuscular hemoglobin concentration (MCHC), optical mean corpuscular hemoglobin concentration CHCM, hemoglobin distribution width (HDW) in g/dL; hematocrit (HCT), red blood cell distribution width (RDW) in %; mean corpuscular volume (MCV), mean platelet volume (MPV) in fL; mean corpuscular hemoglobin (MCH), CH in pg). **(B)** Same as **(A)**, but with ultrasound stimulation. **(C)** Percentage of white blood cells differentials in saline and bacteria instilled animals with sham stimulation (no ultrasound) at 16 hours. **(D)** Same as **(C)**, but after ultrasound stimulation. (WBC, White blood cells; RBC, Red blood cells; HCT, Hematocrit; MCV, Mean corpuscular volume; MCH, Mean corpuscular hemoglobin; MCHC, Mean corpuscular hemoglobin concentration; CHCM, Cellular hemoglobin concentration mean; CH, Cellular hemoglobin; RDW, Red cell distribution width; HDW, Hemoglobin distribution width; MPV, Mean platelet volume; Neut, Neutrophils; Lymph, Lymphocytes; Mono, Monocytes; Eos, Eosinophils; Baso, Basophils; LUC, Large unstained cells). Asterisk indicates *p*<0.05 using non-parametric Wilcoxon rank sum test.

Supplementary Figure 4 Splenic neurotransmitter concentrations in all groups at the 16-hour timepoint (2 hours following ultrasound or sham treatment, i.e., same sample timepoint as transcriptomic/rna sequencing data). **(A–D)** No statistical change in neurotransmitter concentrations were measured for any of the samples at this timepoint. Compared to previous reports (28) the concentrations measured were equivalent to naïve and ultrasound stimulated/CAP activated cohorts (compared to cohorts in which CAP signaling was inhibited by LD_75_ LPS injection (28)).

Supplementary Figure 5 Differential transcript analysis of RNA-Seq blood measures across all treatment groups. Differential analysis was performed on whole blood samples collected from all groups at 18 hours post bacteria/saline instillation and 2 hours post ultrasound stimulation/sham treatment. The Wald test **(A, B)** contrasting bacteria (N=24) vs saline instillations (N=12) resulted in 4,985 transcription changes, 2473 (9% of all transcripts) being upregulated and 2512 (9.1%) being down regulated with p-value < 0.1 adjusted for multiple testing. The Wald test contrasting ultrasound Stimulation (N=18) vs ultrasound Sham (N=18) treatment resulted in only 4 transcript changes with an adjusted p-value < 0.1. Wald test comparisons at the individual group level **(C, D)** were conducted and resulted in 8% less transcriptional changes (2780 vs 3037) in samples receiving ultrasound stimulation vs ultrasound Sham. Wald test comparisons for the Bacteria instilled group had only six differentially expressed transcripts while the Saline instilled group had 222 differentially expressed transcripts (adjusted p-value <0.1).

Supplementary Figure 6
**(A)**. Gene Set Enrichment Analysis results presenting sixteen Gene Ontology (GO) gene sets that were found to be statistically significant (Bonferroni adjusted p value < 0.001) between the bacteria and saline instilled groups for those who receive ultrasound stimulation (left column), ultrasound sham (middle column), and combined groups (right column). **(B)** Heatmap of regulation of inflammatory response (GO:0050727) gene set. **(C)** Heatmap of inflammatory response (GO:0006954) gene set. **(D)** Heatmap of innate immune response (GO:0045087) gene set. **(E)** Heatmap of response to bacterium (GO:0009617) gene set. **(F)** Heatmap of response to type I interferon (GO:0034340) gene set

Supplementary Figure 7 Relative abundances of blood cells in Blood at 18 Hours computed by FARDEEP using the bulk RNA-Seq blood measures and the TIL10 blood cell gene signatures. The cell fractions: **(A)** Tregs; **(B)** T cells CD4; **(C)** Neutrophils; **(D)** B cells; **(E)** Dendritic cells; **(F)** Macrophages M1; **(G)** Macrophages M2; **(H)** T cells CD8; **(I)** Monocytes; **(J)** NK cells are presented for the Saline instilled ultrasound Sham, Saline instilled ultrasound stimulated, Bacteria instilled ultrasound Sham, Bacteria instilled ultrasound stimulated groups.

Supplementary Figure 8 Relative abundances of blood cells in Blood at 18 Hours computed by FARDEEP using the bulk RNA-Seq blood measures and the LM22 blood cell gene signatures. The cell fractions **(A)** Dendritic cells; **(B)** Dendritic cells resting; **(C)** Neutrophils; **(D)** B cells naive; **(E)** B cells memory; **(F)** T cells CD4 naive; **(G)** T cells CD8; **(H)** Eosinophils; (I) Monocytes are presented for the Bacteria instilled vs Saline instilled groups (top row) and for the ultrasound Stimulated vs ultrasound Sham groups (bottom row). The Wilcoxon signed-rank test p-values are Bonferroni corrected for multiple testing.

Supplementary Figure 9 Further analysis of the top differentially expressed M2.Macrophage gene within the TIL10 blood gene signature. **(A)** Re-plot of the M2.Macrophage differentially expressed genes (across the saline/US sham, Saline/US Stim, Bacteria/US Sham, and Bacteria/US Stim cohorts) from the TIL10 gene signature plot within **Figure S6** (the most significant differentially expressed signature between the ultrasound stimulation and sham groups within the signature). **(B)** The top 20 most differentially expressed genes reveals that the majority are involved in promoting either dendritic cell maturation (blue) or monocyte polarization (green) (60–67). **(C)** Plot of the normalized gene counts for the top three differentially expressed genes (RNASE6 (i) (60), SPIB (ii) (61), and INHBA (63) (iii) associated with dendritic cell maturation or monocyte polarization for each of the four cohorts.

Supplementary Figure 10 Peripheral blood mononuclear cells (PBMC) cytokine response assay in controls and bacteria instilled groups. **(A)** TNF-a response in naïve (no challenge) animals to *in-vitro* LPS (10 ng/ml) before after ultrasound stimulation at 16 hours. **(B)** Same as **(A)**, but in bacteria instilled animals at 4 hours, 16 hours, and 48 hours timepoints (Paired t-test). Asterisk indicates *p*<0.05.

Supplementary Figure 11 Hematoxylin and Eosin stain from lung tissues sampled from bacteria infected and saline control rats. **(A)** The lungs were collected during autopsy and sectioned at 20 um thickness using a cryosection machine. The sections were stained with hematoxylin and eosin stain. The left panels show microscopic images of the right lung. The right panels show images of the left lungs. Left lung demonstrates more edema compared to the right lung, which is an indicator of lung injury. **(B)** Gram stain for gram-positive bacteria in the lungs. The lungs were collected during autopsy and sectioned at 20 um thickness using a cryosection machine. The sections were stained with gram positive stain. The left panels show microscopic images of the right lung. The right panels show images of the left lungs. Left lung images demonstrate more gram-positive bacteria compared to the right lung, which indicates localization of the streptococcus pneumonia infection in the left lung.
