# Supplementary material for: Ultrasound Neuromodulation of the Spleen Has Time-Dependent Anti-Inflammatory Effect in a Pneumonia Model
Source: Front Immunol. 2022 Jun 16;13:892086. doi: 10.3389/fimmu.2022.892086 (PMC9244783; doi:10.3389/fimmu.2022.892086)
Supplement: Supplementary Table 1 — Quality metrics for RNA sequencing of blood samples across all three experimental series. The whole blood samples were collected from all groups at 18 hours post bacteria/saline instillation and 2 hours post ultrasound stimulation/sham treatment. The samples were from three different experimental series performed weeks apart between each series. RNA quality and sequencing metrics were of high quality for each sequencing series, with no significant experimental batch effects. RNA integrity numbers (RIN) were >7 for each sample, total number of reads was >30 million for each sample, mean base quality was excellent > 27 (Phred), and percentage of reads mapped to a single location on the reference passed the requirement of > 50%. [file DataSheet_1.pdf]

Supplemental Table 1.

A Quality metrics for the 12 samples from the ultrasound stim series 1

Sample ID	Treatment Group	RNA RIN Score	Total Number of Reads	Mean Base Quality (Phred) score	Uniquely mapped reads %	% of reads unmapped
R86	Saline_Ultrasound	7.2	30,648,255	33.9	63.4%	6.5%
R87	Saline_Ultrasound	7.5	37,569,544	34.0	64.1%	6.1%
R89	Saline_Ultrasound	8.0	31,461,935	34.2	70.1%	4.6%
R95	Saline_Ultrasound	8.2	35,902,759	34.1	60.7%	4.3%
R96	Saline_Ultrasound	7.6	30,862,461	33.8	63.3%	7.9%
R97	Saline_Ultrasound	7.9	34,570,121	33.8	50.6%	9.1%
R99	Bacteria_Ultrasound	n/a	31,899,994	34.0	61.6%	6.3%
R101	Bacteria_Ultrasound	8.5	33,510,350	33.9	60.9%	6.6%
R102	Bacteria_Ultrasound	8.1	32,606,949	34.1	54.5%	5.9%
R105	Bacteria_Ultrasound	7.8	34,729,791	34.0	55.4%	6.3%
R114	Bacteria_Ultrasound	8.0	32,405,966	34.0	62.0%	6.1%
R116	Bacteria_Ultrasound	8.3	36,243,771	34.1	57.2%	7.1%
	Median	8.0	33,058,650	34	61.3%	6.3%

B Quality metrics for the 12 samples from the ultrasound sham series 2

Sample ID	Treatment Group	RNA RIN Score	Total Number of Reads	Mean Base Quality (Phred) score	Uniquely mapped reads %	% of reads unmapped
R197	Saline_Sham	8.2	35,843,488	34.1	55.1%	7.2%
R198	Saline_Sham	7.9	33,624,680	33.9	54.8%	7.9%
R199	Saline_Sham	7.9	38,110,726	33.7	47.8%	10.1%
R200	Saline_Sham	8.0	30,699,406	34.0	54.1%	7.9%
R201	Saline_Sham	8.1	31,484,125	34.1	63.7%	5.6%
R202	Saline_Sham	7.9	33,233,205	33.8	59.6%	8.6%
R192	Bacteria_Sham	8.4	31,376,356	34.1	59.3%	5.1%
R193	Bacteria_Sham	8.2	32,648,367	33.9	58.6%	5.6%
R206	Bacteria_Sham	7.6	34,719,448	34.2	65.0%	4.2%
R207	Bacteria_Sham	8.3	40,319,129	34.2	63.2%	5.0%
R208	Bacteria_Sham	8.2	33,939,937	33.6	65.5%	6.3%
R209	Bacteria_Sham	8.3	34,794,388	33.9	62.8%	7.6%
	Median	8.2	33,782,309	34	59.5%	6.8%

C Quality metrics for the 12 samples from the bacteria series 3

Sample ID	Treatment Group	RNA RIN Score	Total Number of Reads	Mean Base Quality (Phred) score	Uniquely mapped reads %	% of reads unmapped
R219	Bacteria_Sham	8.7	35,580,133	33.8	63.1%	6.1%
R221	Bacteria_Sham	8.8	33,524,986	33.6	68.7%	5.4%
R223	Bacteria_Ultrasound	8.9	35,832,496	33.8	67.1%	6.3%
R224	Bacteria_Ultrasound	9.2	35,255,677	33.8	64.1%	7.1%
R225	Bacteria_Ultrasound	8.7	32,322,015	33.8	66.5%	6.4%
R226	Bacteria_Ultrasound	9.0	38,204,101	33.8	65.7%	5.8%
R227	Bacteria_Ultrasound	9.0	36,309,503	33.7	67.5%	5.1%
R228	Bacteria_Ultrasound	9.3	38,875,664	33.8	66.7%	6.2%
R229	Bacteria_Sham	9.3	31,249,665	33.8	66.3%	6.6%
R230	Bacteria_Sham	9.2	38,481,418	33.8	66.6%	6.2%
R231	Bacteria_Sham	9.1	30,354,705	33.7	65.1%	7.2%
R232	Bacteria_Sham	9.1	31,692,525	33.8	67.8%	6.8%
	Median	9.1	35,417,905	34	66.5%	6.3%

Table S1. Quality metrics for RNA sequencing of blood samples across all three experimental series. The whole blood samples were collected from all groups at 18 hours post bacteria/saline instillation and 2 hours post ultrasound stimulation/sham treatment. The samples were from three different experimental series performed weeks apart between each series. RNA quality and sequencing metrics were of high quality for each sequencing series, with no significant experimental batch effects. RNA integrity numbers (RIN) were >7 for each sample, total number of reads was >30 million for each sample, mean base quality was excellent > 27 (Phred), and percentage of reads mapped to a single location on the reference passed the requirement of > 50%.

Supplemental Figure 1.

S1

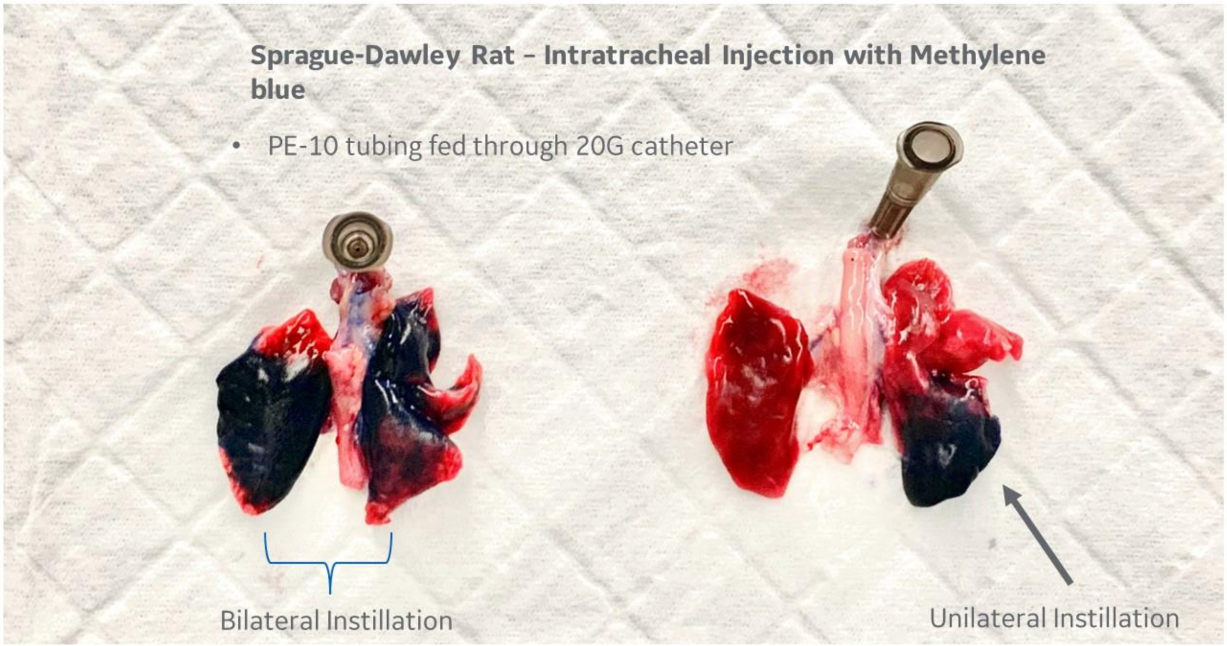

Figure S1. Dye controls used to test the intratracheal injection method. (left) Intratracheal tubes were inserted into both the right and left lung, and methylene blue dye was injected into each side. (right) An Intratracheal tube was inserted into the left lung only, and the methylene blue remained contained within the left lung.

Supplemental Figure S2.

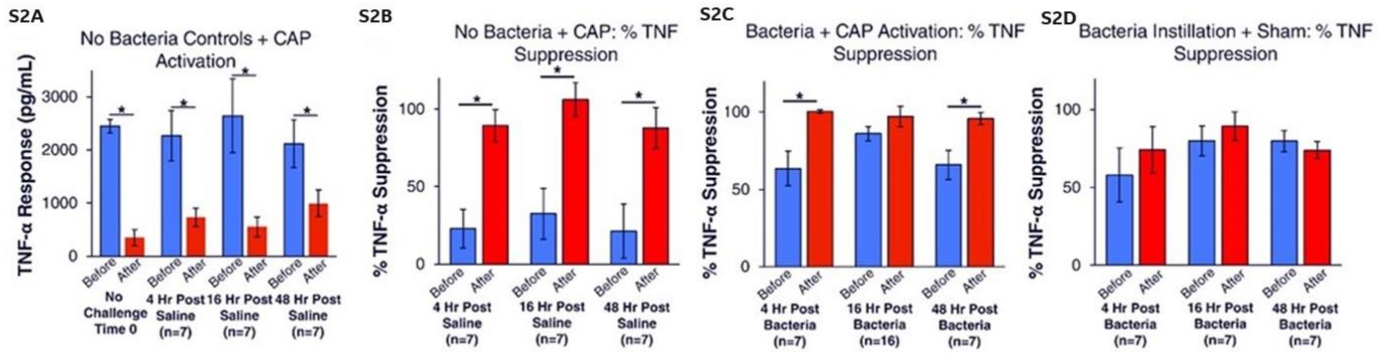

Figure S2. Whole blood Cytokine Response Plotted as Percent Maximal TNF/CAP Suppression. (A) TNF-α response to in-vitro LPS challenge test (10 ng/ml) in instillation naïve animals (left bars) and saline instilled animals (middle and right bars) before and after splenic ultrasound stimulation at multiple timepoints.). (B) % of TNF-α suppression in saline instilled animals. The total change in TNF-α response between pre- and post- ultrasound in naïve (no saline or bacteria instillation) animals (difference between left most blue versus red bars in 2A) was deemed as 100% TNF-α suppression. The pre- and post- ultrasound samples in saline instilled animals at different timepoints were compared with naïve TNF-α suppression and % of TNF-α suppression was calculated. (C) Same as (B), but in bacteria instilled animals with ultrasound stimulation. (D) Same as (B), but in bacteria instilled animals with sham stimulation. Asterisk indicates $p<0.05$ using non-parametric Wilcoxon rank sum test. n=7 for all groups (except bacteria instillation + CAP activation which has n=13).

Supplemental Figure S3.

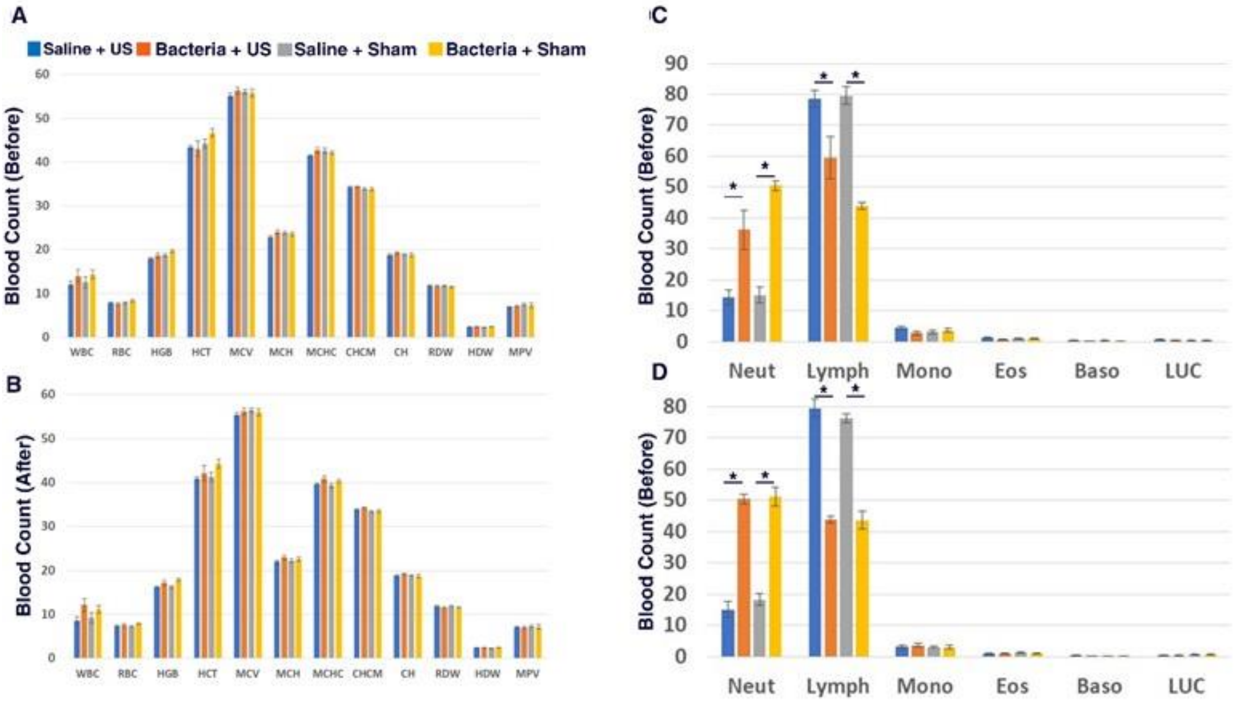

Figure S3. Complete blood count in all groups at the 16 hour timepoint. (A) Complete blood count analysis in saline and bacteria instilled animals with sham stimulation (no ultrasound) at 16 hours. WBC units are represented in 10^3 cells/µL; Red blood cell (RBC) in 10^6 cells/µL; Hemoglobin (HGB), mean corpuscular hemoglobin concentration (MCHC), optical mean corpuscular hemoglobin concentration CHCM, hemoglobin distribution width (HDW) in g/dL; hematocrit (HCT), red blood cell distribution width (RDW) in %; mean corpuscular volume (MCV), mean platelet volume (MPV) in fL; mean corpuscular hemoglobin (MCH), CH in pg). (B) Same as (A), but with ultrasound stimulation. (C) Percentage of white blood cells differentials in saline and bacteria instilled animals with sham stimulation (no ultrasound) at 16 hours. (D) Same as (C), but after ultrasound stimulation. (WBC=White blood cells, RBC=Red blood cells, HCT= Hematocrit, MCV=Mean corpuscular volume, MCH=Mean corpuscular hemoglobin, MCHC=Mean corpuscular hemoglobin concentration, CHCM=Cellular hemoglobin concentration mean, CH=Cellular hemoglobin, RDW=Red cell distribution width, HDW=Hemoglobin distribution width, MPV=Mean platelet volume, Neut=Neutrophils, Lymph=Lymphocytes, Mono=Monocytes, Eos=Eosinophils, Baso=Basophils, LUC=Large unstained cells) Asterisk indicates $p<0.05$ using non-parametric Wilcoxon rank sum test.

Supplemental Figure S4.

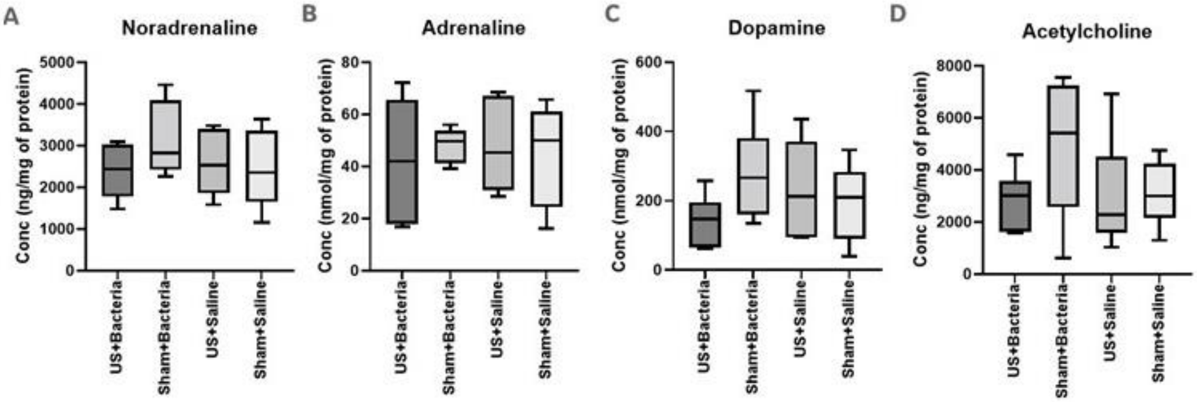

Figure S4. Splenic neurotransmitter concentrations in all groups at the 16-hour timepoint (2 hours following ultrasound or sham treatment, i.e., same sample timepoint as transcriptomic/rna sequencing data). **A-D.** No statistical change in neurotransmitter concentrations were measured for any of the samples at this timepoint. Compared to previous reports[28] the concentrations measured were equivalent to naïve and ultrasound stimulated/CAP activated cohorts (compared to cohorts in which CAP signaling was inhibited by LD_{75} LPS injection[28]).

Supplemental Figure S5.

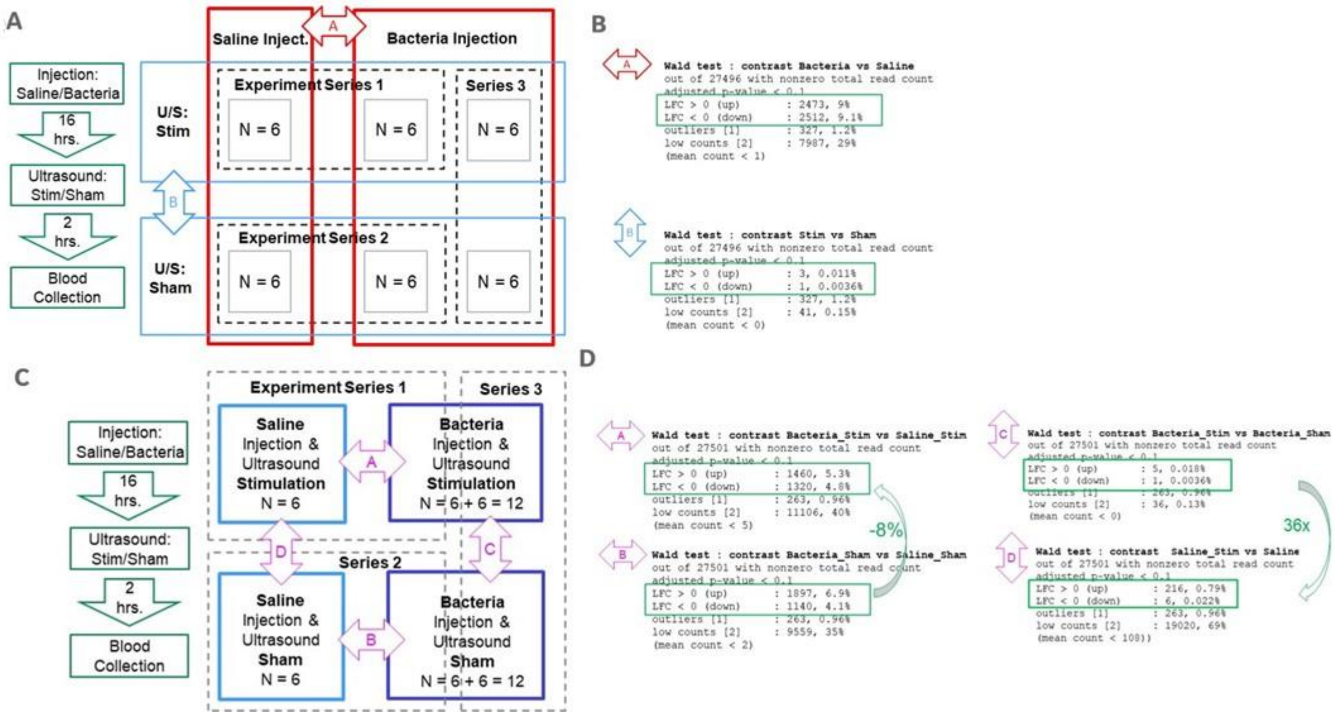

Figure S5. Differential transcript analysis of RNA-Seq blood measures across all treatment groups. Differential analysis was performed on whole blood samples collected from all groups at 18 hours post bacteria/saline instillation and 2 hours post ultrasound stimulation/sham treatment. The Wald test (A & B) contrasting bacteria (N=24) vs saline instillations (N=12) resulted in 4,985 transcription changes, 2473 (9% of all transcripts) being upregulated and 2512 (9.1%) being down regulated with p-value < 0.1 adjusted for multiple testing. The Wald test contrasting ultrasound Stimulation (N=18) vs ultrasound Sham (N=18) treatment resulted in only 4 transcript changes with an adjusted p-value < 0.1. Wald test comparisons at the individual group level (C-D) were conducted and resulted in 8% less transcriptional changes (2780 vs 3037) in samples receiving ultrasound stimulation vs ultrasound Sham. Wald test comparisons for the Bacteria instilled group had only six differentially expressed transcripts while the Saline instilled group had 222 differentially expressed transcripts (adjusted p-value <0.1).

Supplemental Figure S6A.

NAME	Ultrasound Treated Group BacteriaStimvsSalineStim.FWER.p.val	U/S Sham Treated GroupBacteriaShamvsSalineSham.FWER.p.val	BacteriavsSaline.FWER.p.val
GO_REGULATION_OF_INFLAMMATORY_RESPONSE	0.001	0.001	0.001
GO_INFLAMMATORY_RESPONSE	0.001	0.001	0.001
GO_INNATE_IMMUNE_RESPONSE	0.001	0.001	0.001
GO_RESPONSE_TO_BACTERIUM	0.001	0.001	0.001
GO_RESPONSE_TO_TYPE_I_INTERFERON	0.001	0.001	0.001
GO_DEFENSE_RESPONSE	0.001	0.001	0.001
GO_DEFENSE_RESPONSE_TO_BACTERIUM	0.001	0.001	0.001
GO_DEFENSE_RESPONSE_TO_OTHER_ORGANISM	0.001	0.001	0.001
GO_DETECTION_OF_BIOTIC_STIMULUS	0.001	0.002	0.001
GO_RESPONSE_TO_MOLECULE_OF_BACTERIAL_ORIGIN	0.001	0.003	0.001
GO_DEFENSE_RESPONSE_TO_VIRUS	0.001	0.168	0.001
GO_POSITIVE_REGULATION_OF_INTERLEUKIN_6_PRODUCTION	0.01	0.001	0.001
GO_POSITIVE_REGULATION_OF_INFLAMMATORY_RESPONSE	0.035	0.001	0.001
GO_REGULATION_OF_RESPONSE_TO_WOUNDING	0.005	0.001	0.001
GO_LEUKOCYTE_CHEMOTAXIS	0.002	0.078	0.001
GO_REGULATION_OF_ACUTE_INFLAMMATORY_RESPONSE	0.356	0.003	0.001

Figure S6A. Gene Set Enrichment Analysis results presenting sixteen Gene Ontology (GO) gene sets that were found to be statistically significant (Bonferroni adjusted p value < 0.001) between the bacteria and saline instilled groups for those who receive ultrasound stimulation (left column), ultrasound sham (middle column), and combined groups (right column).

Supplemental Figure S6B.

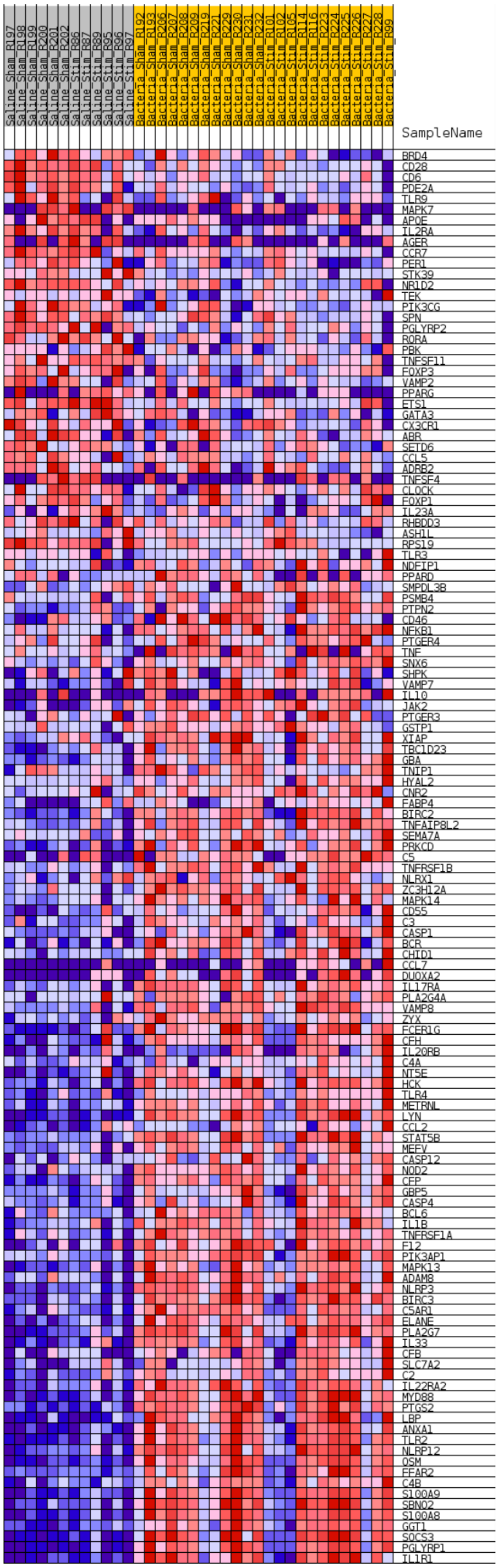

Figure S6B. Heatmap of regulation of inflammatory response (GO:0050727) gene set

Supplemental Figure S6C.

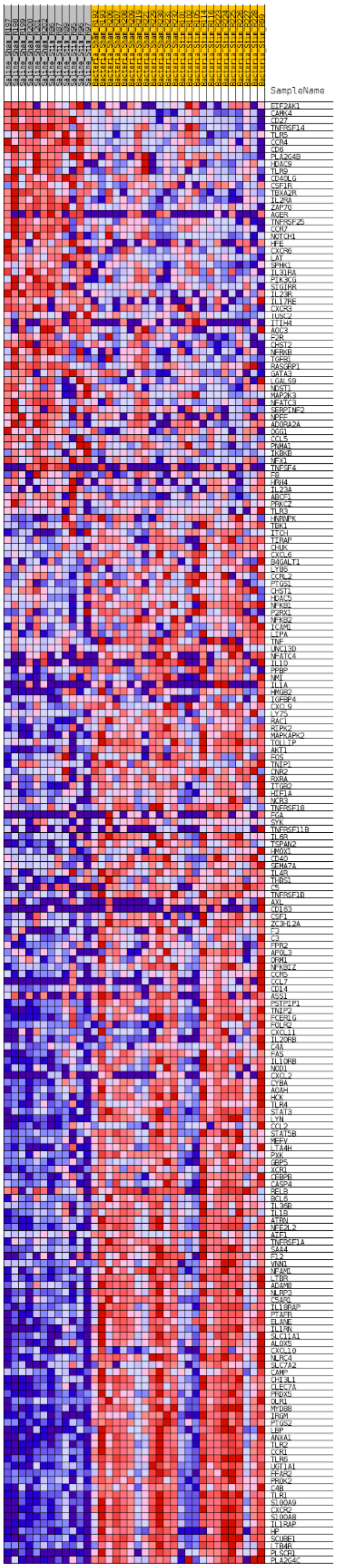

Figure S6C. Heatmap of inflammatory response (GO:0006954) gene set

Supplemental Figure S6D.

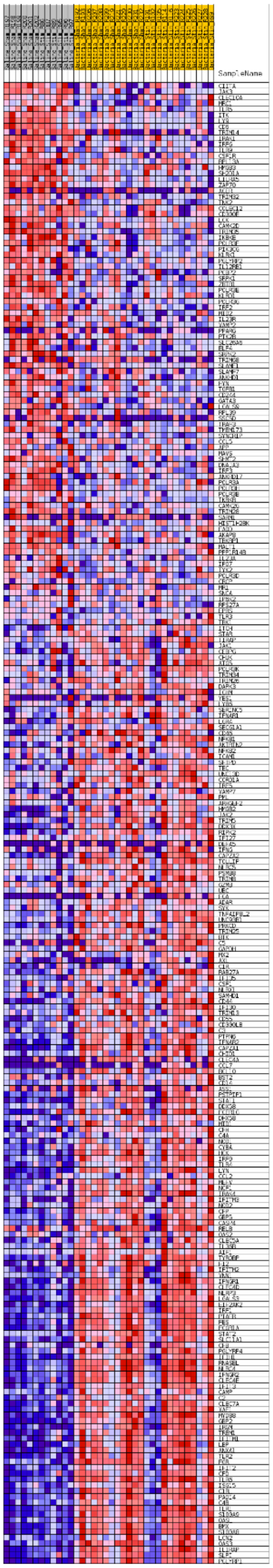

Figure S6D. Heatmap of innate immune response (GO:0045087) gene set

Supplemental Figure S6E.

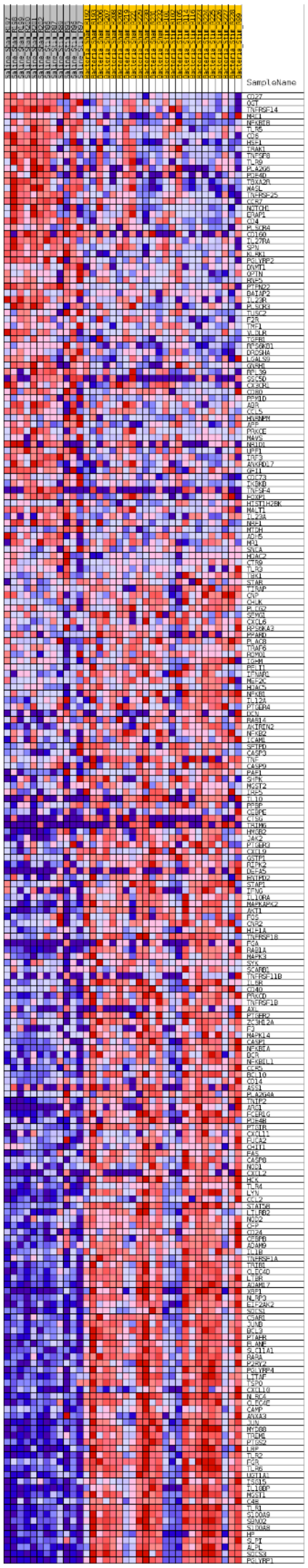

Figure S6E. Heatmap of response to bacterium (GO:0009617) gene set

Supplemental Figure S6F.

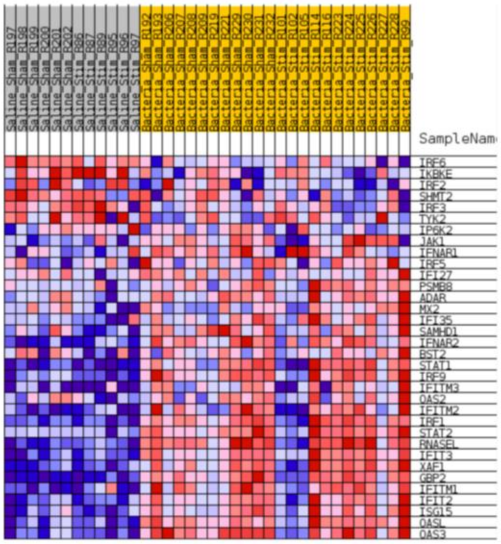

Figure S6F. Heatmap of response to type I interferon (GO:0034340) gene set

Supplemental Figure S7.

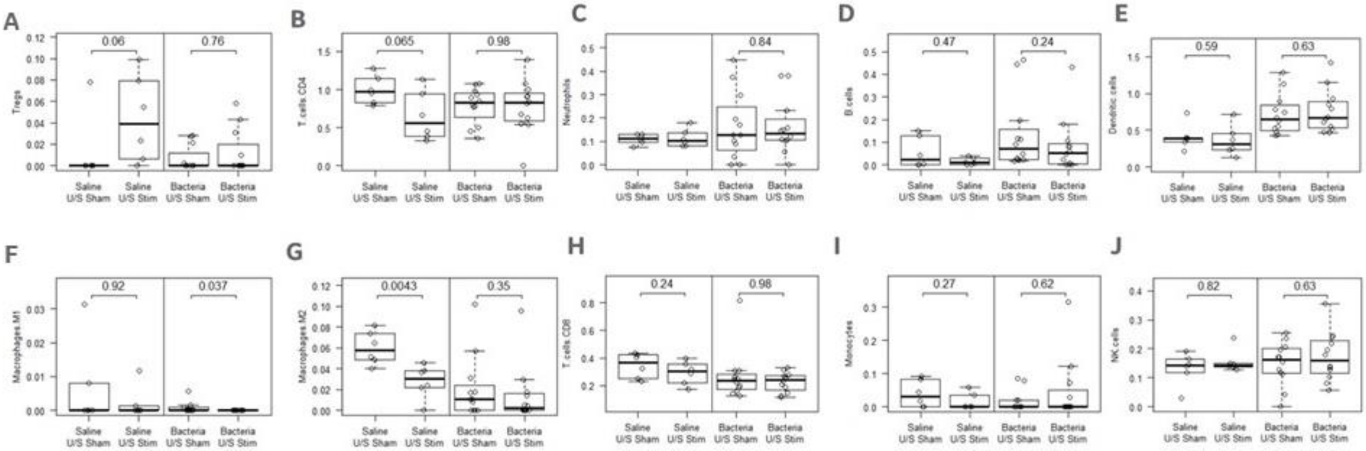

Figure S7. Relative abundances of blood cells in Blood at 18 Hours computed by FARDEEP using the bulk RNA-Seq blood measures and the TIL10 blood cell gene signatures. The cell fractions: (A) Tregs; (B) T cells CD4; (C) Neutrophils; (D) B cells; (E) Dendritic cells; (F) Macrophages M1; (G) Macrophages M2; (H) T cells CD8; (I) Monocytes; (J) NK cells are presented for the Saline instilled ultrasound Sham, Saline instilled ultrasound stimulated, Bacteria instilled ultrasound Sham, Bacteria instilled ultrasound stimulated groups.

Supplemental Figure S8.

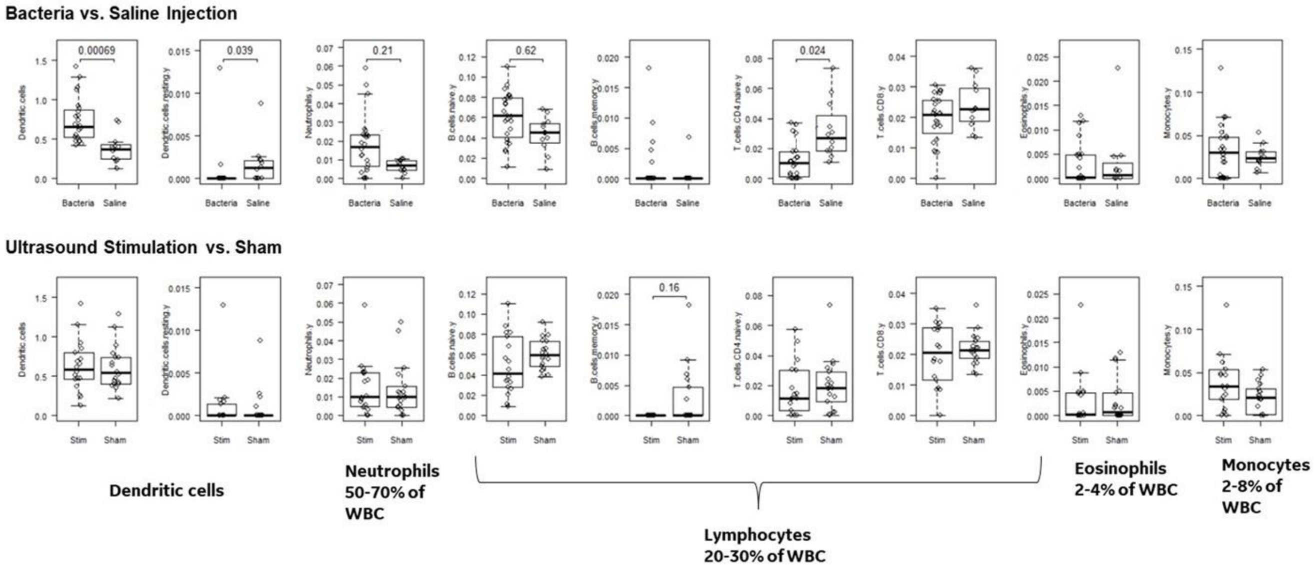

Figure S8. Relative abundances of blood cells in Blood at 18 Hours computed by FARDEEP using the bulk RNA-Seq blood measures and the LM22 blood cell gene signatures. The cell fractions (A) Dendritic cells; (B) Dendritic cells resting; (C) Neutrophils; (D) B cells naive; (E) B cells memory; (F) T cells CD4 naive; (G) T cells CD8; (H) Eosinophils; (I) Monocytes are presented for the Bacteria instilled vs Saline instilled groups (top row) and for the ultrasound Stimulated vs ultrasound Sham groups (bottom row). The Wilcoxon signed-rank test p-values are Bonferroni corrected for multiple testing.

Supplemental Figure S9

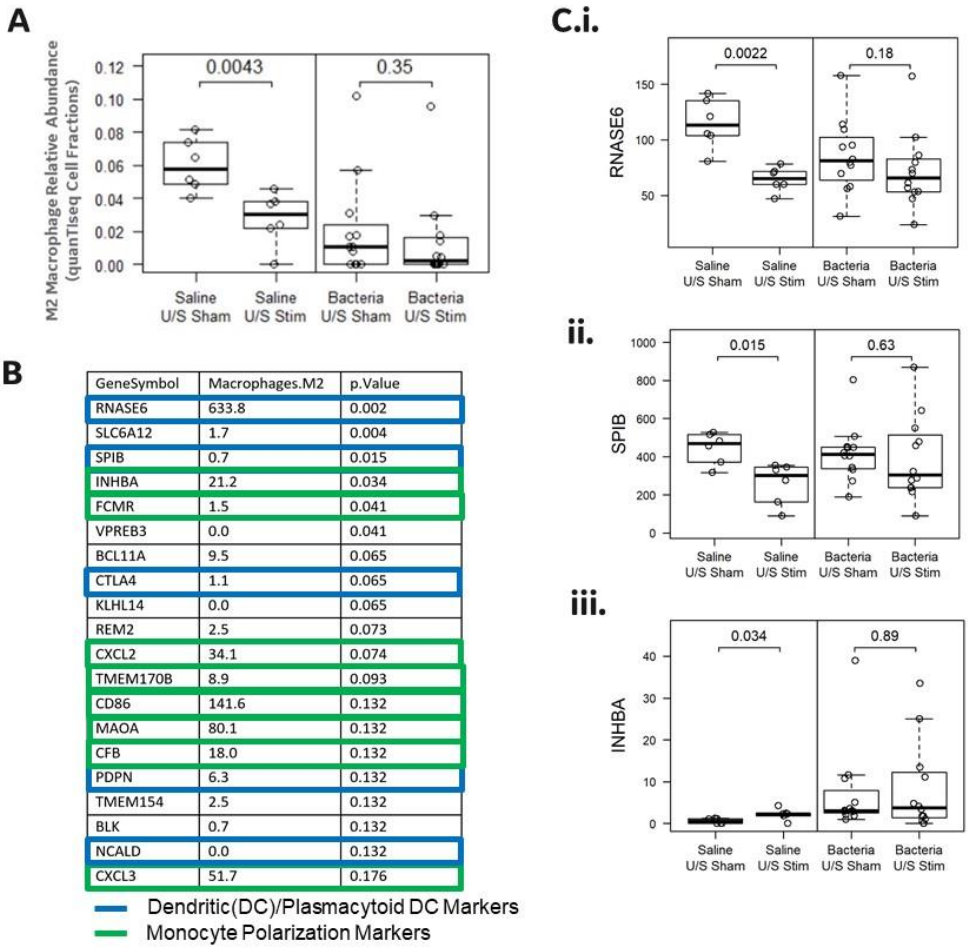

GeneSymbol	Macrophages.M2	p.Value
RNASE6	633.8	0.002
SLC6A12	1.7	0.004
SPIB	0.7	0.015
INHBA	21.2	0.034
FCMR	1.5	0.041
VPREB3	0.0	0.041
BCL11A	9.5	0.065
CTLA4	1.1	0.065
KLHL14	0.0	0.065
REM2	2.5	0.073
CXCL2	34.1	0.074
TMEM170B	8.9	0.093
CD86	141.6	0.132
MAOA	80.1	0.132
CFB	18.0	0.132
PDPN	6.3	0.132
TMEM154	2.5	0.132
BLK	0.7	0.132
NCALD	0.0	0.132
CXCL3	51.7	0.176

Figure S9. Further analysis of the top differentially expressed M2.Macrophage gene within the TIL10 blood gene signature . A. Re-plot of the M2.Macrophage differentially expressed genes (across the saline/US sham, Saline/US Stim, Bacteria/US Sham, and Bacteria/US Stim cohorts) from the TIL10 gene signature plot within figure S6 (the most significant differentially expressed signature between the ultrasound stimulation and sham groups within the signature). B. The top 20 most differentially expressed genes reveals that the majority are involved in promoting either dendritic cell maturation (blue) or monocyte polarization (green).[60–67] C. Plot of the normalized gene counts for the top three differentially expressed genes (RNASE6 (i)[60], SPIB (ii)[61], and INHBA[63] (iii) associated with dendritic cell maturation or monocyte polarization for each of the four cohorts.

Supplemental Figure S10.

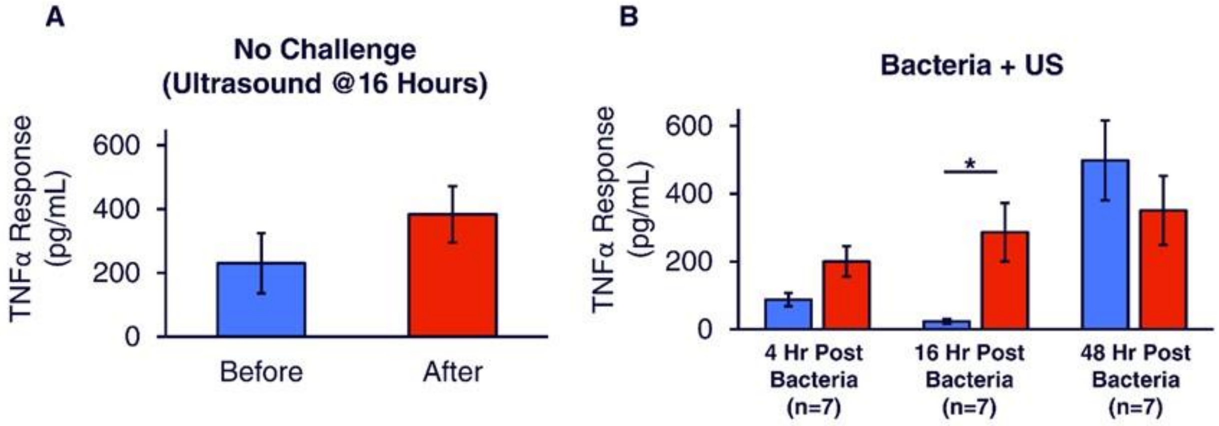

Figure S10. Peripheral blood mononuclear cells (PBMC) cytokine response assay in controls and bacteria instilled groups. (A) TNF-a response in naïve (no challenge) animals to in-vitro LPS (10 ng/ml) before after ultrasound stimulation at 16 hours. (B) Same as (A), but in bacteria instilled animals at 4 hours, 16 hours, and 48 hours timepoints (Paired t-test). Asterisk indicates $p<0.05$.

Supplemental Figure S11

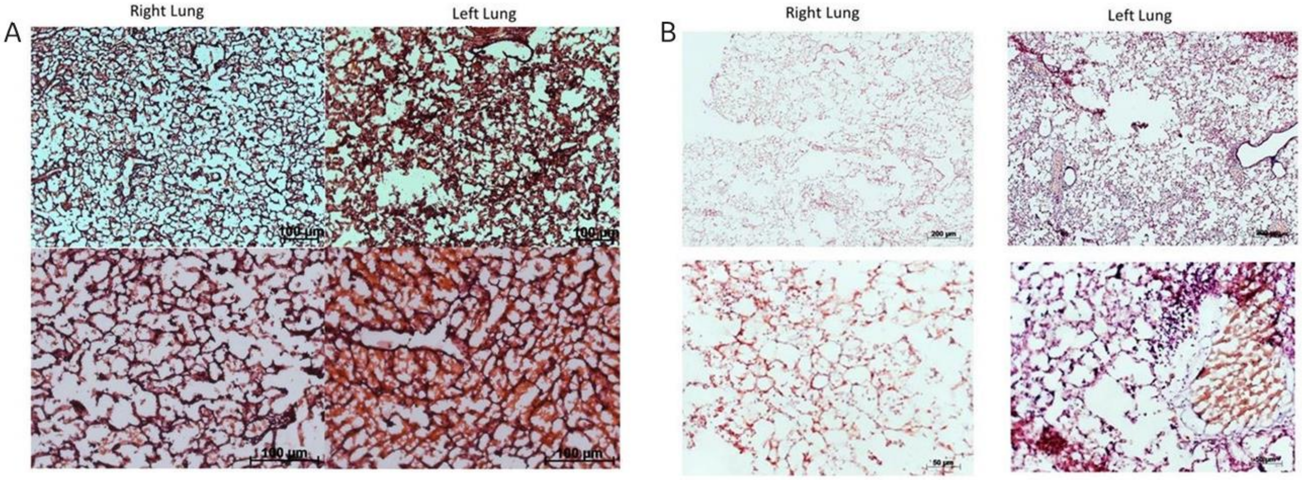

Figure S11. Hematoxylin and Eosin stain from lung tissues sampled from bacteria infected and saline control rats. A. The lungs were collected during autopsy and sectioned at 20 um thickness using a cryosection machine. The sections were stained with hematoxylin and eosin stain. The left panels show microscopic images of the right lung. The right panels show images of the left lungs. Left lung demonstrates more edema compared to the right lung, which is an indicator of lung injury. B. Gram stain for gram-positive bacteria in the lungs. The lungs were collected during autopsy and sectioned at 20 um thickness using a cryosection machine. The sections were stained with gram positive stain. The left panels show microscopic images of the right lung. The right panels show images of the left lungs. Left lung images demonstrate more gram-positive bacteria compared to the right lung, which indicates localization of the streptococcus pneumonia infection in the left lung.
